# Signatures of EMT, immunosuppression, and inflammation in primary and recurrent human cutaneous squamous cell carcinoma at single-cell resolution

**DOI:** 10.7150/thno.77528

**Published:** 2022-10-31

**Authors:** Xin Li, Shuang Zhao, Xiaohui Bian, Lining Zhang, Lixia Lu, Shiyao Pei, Liang Dong, Wensheng Shi, Lingjuan Huang, Xiyuan Zhang, Mingliang Chen, Xiang Chen, Mingzhu Yin

**Affiliations:** 1 Department of Dermatology, Hunan Engineering Research Center of Skin Health and Disease, Hunan Key Laboratory of Skin Cancer and Psoriasis, Xiangya Hospital, Central South University, Changsha, Hunan 410008, China.; 2 National Engineering Research Center of Personalized Diagnostic and Therapeutic Technology, Central South University, Changsha, Hunan 410008, China.; 3 Department of Urology, Xiangya Hospital, Central South University, Changsha, Hunan 410008, China.

**Keywords:** cSCC, recurrence, TME, EMT, immunosuppression, inflammation

## Abstract

**Rationale:** The recurrence of cutaneous squamous cell carcinoma (cSCC) after surgery is associated with the reprogramming of the tumor microenvironment (TME), and remains a key factor affecting its outcomes.

**Methods:** We employed single-cell RNA sequencing (scRNA-seq) to examine the dynamic changes in epithelial cells, T cells, myeloid cells, and fibroblasts between primary and recurrent cSCC. Cell clustering, cell trajectory, cell-cell communication, and gene set enrichment analysis were used to investigate the TME heterogeneity between primary and recurrent cSCC. Gene expression differences were monitored by IHC staining.

**Results:** We examined the immunosuppressed microenvironment in recurrent cSCC, which exhibited a T cell-excluded and SPP1^+^ tumor-associated macrophages (TAMs)-enriched status. In recurrent cSCC, CD8^+^ T cells showed high exhaustion and low inflammatory features, while SPP1^+^ TAMs displayed global pro-tumor characteristics, including decreased phagocytosis and inflammation and increased angiogenesis. Furthermore, the subgroups of SPP1^+^ TAMs harbored distinct functions. SPP1^+^ CD209^high^ TAMs showed features of phagocytosis, while SPP1^+^ CD209^low^ TAMs tended to have a high angiogenic ability. A subpopulation of tumor-specific keratinocytes (TSKs) showed significant epithelial-mesenchymal transition (EMT) features in recurrent cSCC, probably due to their active communication with IL7R**^+^** cancer-associated fibroblasts (CAFs). Moreover, we found that the pleiotropic growth factor/cytokine Midkine (MDK) could provoke different cell-cell interactions in cSCC with distinctive staging. In primary cSCC, MDK was highly expressed in fibroblasts and could promote their proliferation and block the migration of tumor cells, while in recurrent cSCC, the high expression of MDK in TSKs promoted their proliferation and metastasis.

**Conclusion:** Our study provides insights into the critical mechanisms of cSCC progression, which might facilitate the development of a powerful approach for the prevention and treatment of cSCC recurrence.

## Introduction

Cutaneous squamous cell carcinoma (cSCC) remains the second most common nonmelanoma skin cancer after basal cell carcinoma (BCC) 1. It originates from epidermal keratinocytes and can develop as an *in situ*, invasive, and finally metastatic form 2, 3. Although most cases of cSCC can be completely eradicated by surgery or ablation, a fraction of these tumors recur and metastasize, leading to death 4, and are considered high-risk tumors 5. Therefore, there is an urgent need to distinguish high-risk cSCCs and prevent their progression in the early stage, which could significantly improve the morbidity and mortality of cSCC. The immunosuppressed phenotype has been observed as an important factor for cSCC recurrence 6. Thus, it is worthwhile to characterize the heterogeneous and functional states of cells in the tumor microenvironment (TME) of cSCC.

The TME is composed of highly plastic cancer cells, resident and infiltrating host cells, and noncellular tissue components, which constantly interact and collectively determine tumor progression, metastasis, and therapeutic responses 7, 8. Recently, single-cell RNA sequencing (scRNA-seq) technology has enabled an unprecedented understanding of intra-tumoral transcriptomic heterogeneity in many cancers 9, revealing critical cell populations that influence drug resistance and tumor prognosis 10, 11. A recent study utilized scRNA-seq to dissect the tumor and immune dynamics of cSCC and depicted a global ligand-receptor association of primary human cSCC and matched normal skin 12. However, the heterogeneity between primary and recurrent cSCCs and the pathogenesis of cSCC recurrence are not fully understood.

We performed scRNA-seq in 5 patients diagnosed with primary or recurrent cSCC to comprehensively analyze the characteristics and alterations of the cSCC TME. In total, 14,626 single-cell transcriptomes were characterized from tumor tissues and adjacent normal skin (ANS) sites. We identified a subset of tumor-specific keratinocytes (TSKs) with remarkable epithelial-mesenchymal transition (EMT) features in recurrent cSCC. Extending the findings in previous work 13, we observed two subpopulations of SPP1^+^ tumor-associated macrophages (TAMs) characterized by high phagocytosis (SPP1^+^ CD209^high^ TAMs) and angiogenesis (SPP1^+^ CD209^low^ TAMs) abilities. The primary and recurrent cSCCs displayed strikingly distinct compositions of immune cells and fibroblasts, with recurrent cSCC showing a T-cell-excluded and SPP1^+^ TAM-enriched microenvironment. In recurrent cSCC, T cells exhibited high exhaustion and a low inflammatory state, while TAMs displayed low phagocytosis, inflammatory features, and high angiogenic ability. We also identified a group of cancer-associated fibroblasts (CAFs) enriched in recurrent cSCC that closely interacted with TSKs. Finally, we found that the pleiotropic growth factor/cytokine Midkine (MDK) was upregulated in fibroblasts of primary tumors and potentially promoted their proliferation, further blocking the migration of tumor cells from the TME. Based on the IHC staining, MDK expression was upregulated in recurrent cSCC and was positively correlated with two EMT-associated genes, VIM and TGFB1, possibly playing a critical role in facilitating the proliferation and metastasis of tumor cells. Our study emphasizes the potential role of MDK in regulating the cell-cell crosstalk between primary and recurrent cSCC that may serve as a key factor in mediating cSCC recurrence.

## Methods

### Patients and sample collection

Five patients that pathologically diagnosed with cSCC at Xiangya Hospital of Central South University were investigated in this study, including four primary cSCC patients and one recurrent cSCC patient. Fresh tumor and adjacent skin samples from primary and recurrent cSCC were surgically resected from the above-described patients (Table S1). All subjects provided written informed consent, and this study was approved by the institutional ethics committee of Xiangya Hospital of Central South University (2022020109).

### Preparation of single-cell suspensions

The fresh tissues were stored in the MACS Tissue storage solution (Miltenyi Biotec, 130-100-008) on ice after the surgery within 30 mins, and washed with Hanks Balanced Salt Solution (HBSS) for three times before dissociation. The Human Tumor Dissociation Kit (Miltenyi Biotec, 130-095-929), gentle MACS Dissociator (Miltenyi Biotec, 130-093-235) and gentle MACS C Tubes (Miltenyi Biotec, 130-093-237) were used for obtaining single-cell suspensions, Dead Cell Removal Kit (Miltenyi Biotec, 130-090-101) was used to improve cell viability to meet the requirements of single cell sequencing.

### Single-Cell RNA sequencing and Library preparation

BD Rhapsody system and Singleron platform were used in our study.

#### Sequencing with Singleron platform

Single-cell suspensions (1×10^5^ cells/ml) with PBS (HyClone) were loaded into microfluidic devices using the Singleron Matrix^®^ Single Cell Processing System (Singleron). Subsequently, the scRNA-seq libraries were constructed according to the protocol of the GEXSCOPE^®^ Single Cell RNA Library Kits (Singleron) 14. Individual libraries were diluted to 4 nM and pooled for sequencing. At last, pools were sequenced on Illumina HiSeq X with 150 bp paired end reads.

#### Sequencing with BD Rhapsody system

Cells from each patient were labeled with sample tags from the Human Immune Single-Cell Multiplexing Kit (BD Biosciences) according to the manufacturer's instruction. The cell capture beads were retrieved for reverse transcription as per the manufacturer's protocol (BD Biosciences, Single-Cell Capture and cDNA Synthesis). Single-cell capture and cDNA library preparation were performed using the BD Rhapsody Express Single-Cell Analysis System (BD Biosciences), following the manufacturer's instructions. The libraries were loaded on an S1 flow cell (2 ×100 cycle) and paired-end sequenced at >200,000 reads per cell depth on a Novaseq 6000 Sequencer (Illumina) at the Kinghorn Centre for Cellular Genomics, Garvan Institute, Sydney, Australia. PhiX (20%) was added to the sequencing run to compensate for the low complexity library 15.

### Raw data processing of single-cell RNA sequencing data

For data generated with Singleron platform, reads were mapped to the human genome (GRCh38) using scopetools (https://github.com/SingleronBio/SCOPE-tools). First, cell barcode and unique molecular identifier (UMI) were extracted after filtering read one according to the sequence information, the corrected barcode and original UMI sequence were added to the ID of read two. After that, read two were trimmed by cutadapt and then aligned to the reference genome using STAR 16, 17. Furthermore, featureCounts was applied to target reads to the genomic position of genes (ensemble version 99) 18. Finally, reads with the same cell barcode, UMI and gene were grouped together to count the number of UMIs per gene per cell.

For data generated with BD platform, the FASTQ files were processed following the BD Rhapsody Analysis pipeline (BD Biosciences), which was implemented in the CWL-runner. Briefly, read pairs with low quality were removed, the quality-filtered R1 reads were analysed to identify cell label and UMI sequences. Next, the pipeline used STAR to map the filtered R2 reads to the transcriptome. Reads with the same cell label, the same UMI sequence and the same gene were collapsed into a single raw molecule. The obtained counts were adjusted by BD Biosciences-developed error correction algorithm—recursive substitution error correction (RSEC) to correct sequencing and PCR errors. Barcoded oligo-conjugated antibodies (single-cell multiplexing kit; BD Biosciences) were used to infer the origin of sample by the BD Rhapsody Analysis pipeline.

### Quality control, batch correction, and major cell type annotation of single-cell RNA sequencing data

The UMI count data of Singleron and BD platform were imported into Seurat (V4.1.0). The following initial cell filtering steps were performed: 1) cells with more than 15% mitochondrial counts were removed; 2) cells expressing less than 200 genes were removed; 3) cells expressing more than 25,000 UMIs were removed. Putative doublets were removed for each sample using the Scrublet tool with the default parameters 19. Single-cell gene expression data was normalized using the “LogNormalize” method with a scale factor 10,000. Next, the top 2,000 most variable genes were identified and a linear scaling method was applied to standardize the range of expression values for each gene. Principal component analysis (PCA) was performed to reduce dimensionality by “RunPCA” function. The top 50 principal components (PCs) were used for Uniform Manifold Approximation and Projection for dimension reduction (UMAP). To integrate cells from different samples and platforms for unsupervised clustering, we used harmony and set sample and platform as two technical covariates for batch correction 20. Cell clusters were identified using the “FindClusters” function, with the resolution of 0.04. The most significant differentially expressed genes (DEGs) in each cluster were identified using the “FindAllMarker” function, using the Wilcoxon's test. We further identified major cell types according to the gene expression of well-known markers: Epithelial cells: KRT14, KRT5; T cells: CD2, CD3D; Fibroblasts: COL1A1, DCN; Myeloid cells: LYZ, HLA-DRA; Endothelial cells: RAMP2, VWF; Mast cells: CPA3, KIT; B cells: MZB1, CD79A.

### Sub-clustering of major cell types

For the epithelial cells, T cells, fibroblasts and myeloid cells, we extracted cells from the integrated dataset for sub-clustering. Gene re-scaling, dimensionality reduction, batch correction and cell clustering were performed as described above.

For epithelial cells, we examined the following well-known markers and divided them into 4 cell types: Cycling cells: MKI67, TOP2A; TSKs: PTHLH, MMP10; Basal cells: COL17A1; Differentiating cells: KRT1, KRT16. The Cycling cells were further clustered into cycling basal cells and cycling TSKs based on the above markers. Specifically, we removed cell clusters that highly expressed the cell markers of Pilosebaceous/Eccrine (SAA1, LHX2), fibroblasts (COL1A1), T cells (CD2, CD3D), Mast cells (TPSAB1, TPSB2) and Myeloid cells (LYZ), which were regarded as doublets (Supplementary Figure 2). Few cells (2 cells) from the ANS were clustered into TSK, which may be caused by sample contamination, we thus removed these cells in the downstream analysis.

For T cells, cell subpopulations were determined according to the following gene markers: Effector CD4 T cells: CD4; Naive CD4^+^ T cells: CD4, CCR7, SELL; Effector CD8 T cells: CD8A, GZMA; CD8^+^ cytotoxic cells: CD8, IFNG, GZMA; Tregs: CD4, IL2RA, FOXP3, CTLA4.

The myeloid cells were sub-clustered based on the gene expression of the following markers: Monocytes: VEGFA, VCAN, FCN1; SPP1^+^ CD209^high^ TAMs: SPP1, CD209^high^, CD163, MRC1, CCL18; SPP1^+^ CD209^low^ TAMs: SPP1, CD209^low^, CD163, MRC1, CCL18; CXCL10^+^ TAMs: CD163, MRC1, CXCL10; Cycling TAMs: CD209, CD163, MRC1, TOP2A, MKI67; CD14^+^ dendritic cells (DCs): CD1A, CD14, CD1C; CLEC9A^+^ DCs: CLEC9A, CD1C; CD1a^+^ CD1c^+^ DCs: CLEC10A, CD1A, CD1C; CXCL9-11^+^ MDSCs: CXCL9, CXCL10, CXCL11, IL1B, S100A8, S100A9; CXCL1-3^+^ MDSCs: CXCL1, CXCL2, CXCL3, IL1B, S100A8, S100A9.

For fibroblasts, cell subpopulations were determined according to the gene expression of the following markers: mCAFs: RGS5, DCN, COL6A2, COL1A1, COL1A2; iCAFs: RGS5, DCN, COL6A2, COL1A1, COL1A2; IL7R**^+^** CAFs: IL7R, IL1B, IL6, CXCL1, CXCL3, CXCL5, CXCL6, CXCL8, CXCL13, CXCL14, DCN, COL6A2, COL1A1, COL1A2.

### Correlation analysis between different cell types

To explore the correlation between different cell types, we first calculated the mean gene expression level of cells that belong to the same cell type, and merged them to computed spearman correlation coefficient. Pheatmap package (V1.0.12) was used to visualized the correlation coefficient between different cell types.

### Identification of differentially expressed genes

The “FindMarkers” function in Seurat package was used to detect differentially expressed genes, we defined genes with adjusted *P* value < 0.05, average log2FC > 1 as up-regulated genes and genes with adjusted *P* value < 0.05, average log2FC < -1 as down-regulated genes.

### Cell-cell communication analysis

The CellChat (V1.1.3, https://github.com/sqjin/CellChat) algorithm was used to infer cell-cell interactions within TME and identify differential interactions between primary and recurrent samples 21. In brief, we followed the official workflow and imported gene expression data of cSCC into CellChat using “createCellChat” function. We mainly applied “identifyOverExpressedGenes”, “identifyOverExpressedInteractions”, “projectData” functions to detect significant cell-cell interactions among the investigated cells. The “compareInteractions”, “RankNet” functions were used to perform interaction comparison between primary and recurrent samples. The “netAnalysis_signalingRole_scatter” function was used to calculate the incoming and outgoing interaction strengthen of cells among datasets. All cell interaction visualizations were plotted using the CellChat package.

### Gene set variation analysis

#### Hallmarks of cancer activity analysis

We collected GO terms mapping to the hallmarks of cancer 22, which were used to evaluate the tumor property of TSKs. We applied GSVA method to calculate the hallmark score of individual cells, as implemented in the GSVA R package (V1.40.1) 23.

#### Pathway and gene signature score analysis

We obtained genes from four pathways derived from Kyoto Encyclopedia of Genes and Genomes (KEGG) to evaluated the inflammatory score: TNF signaling pathway (hsa04668), NF-kappa B signaling pathway (hsa04064), IL-17 signaling pathway (hsa04657) and NOD-like receptor signaling pathway (hsa04621). Besides, genes in cMAP signaling pathway (hsa04024) were used to calculate cMAP activity score. We also evaluated the phagocytosis score of myeloid cells by collecting its associated genes from KEGG (hsa04666). The EMT signature related genes were downloaded from MSigDB 24, which were used to evaluated the EMT score of TSKs. The score of above-mentioned pathways or signature was also calculated using GSVA R package (V1.40.1). To compare the differences of scores between different sample or cell types, we used the Wilcoxon signed-rank test that implemented in the ggpubr package (V0.4.0) to perform significance tests.

### Gene set enrichment analysis

We performed KEGG enrichment analysis using the differentially expressed genes by clusterProfiler (V4.1.4) package 25, 26. Pathways with *Q* value < 0.05 were regarded as significant enriched results. Besides, we also did gene set enrichment analysis (GSEA) of KEGG pathways using clusterProfiler package, a cutoff *Q* value < 0.05 was applied to select the most significantly enriched pathways.

### Definition of exhaustion score for CD8^+^ T cells

To evaluate the exhaustion status of CD8^+^ T cells in our study, we used a group of exhaustion-related genes to define the exhaustion score 10. Specifically, the exhaustion score was defined as the average expression of CXCL13, HAVCR2, PDCD1, TIGIT, LAG3, CTLA4, LAYN, RBPJ, VCAM1, TOX and MYO7A. Wilcoxon signed-rank test was used to perform significance tests, which was implemented in the ggpubr package (V0.4.0).

### Construction of single-cell trajectories

#### RNA velocity estimation

The bam files generated by Singleron and BD Rhapsody Analysis pipeline were imported into the Velocyto pipeline 27 to generate loom files, which recorded the count matrices for spliced and unspliced reads. The resulting loom files were fed into the scVelo package (V0.2.4) 28 to computed steady-state gene-specific velocities, the final cell transition status was visualized in our original UMAP embedding.

#### CytoTRACE analysis

We performed CytoTRACE 29 analysis with default parameters following the official guidance, which could predict cell differentiation states from scRNA-seq data. The CytoTRACE score was used to verify the trajectory analysis from scVelo package.

### IHC staining in human cSCC samples

For immunohistochemical staining, samples were sectioned for 4 um. Tissue sections heated (65°C) in an oven for 2 hours, followed by 40 minutes in deparaffinization and rehydrated with graded alcohol concentrations using standard procedures. Immersed in the already boiling 10nM citric acid (pH 6.0), the deparaffinized sections heated on low heat for 20 minutes in a microwave oven. For blocking endogenous peroxidase activity, the deparaffinized sections was incubated with endogenous peroxidase blocking solution for 10 minutes, and then incubated with normal goat serum for blocking for 1 hour to block nonspecific immunoglobulin binding. Then, the slides were incubated at 4°C overnight with a primary rabbit monoclonal antibody against MDK (1:100, ab52637, Abcam), VIM (1:200, TU253239, abmart), TGFB1 (1:200, PU159710, abmart). The next day, the slides were incubated with IHC enhancer for 20 minutes, followed by incubation with the corresponding secondary antibody conjugated with horseradish peroxidase at 37℃ for 1 hour. Next, DAB (3,3-diaminodbenzidine) substrate was added, and then counterstained with hematoxylin for 5 min. Finally, the sections were dehydrated, cleared and mounted in aqueous mounting medium for microscopic evaluation.

### Semi-quantitative analysis of IHC staining

To achieve high quality results, two independent pathologists experienced in evaluating IHC participated in reviewing samples, who were blinded to the clinical outcome of these patients. We assessed the percentage of positively stained immuno-reactive cells and the staining intensity to semi-quantitatively determine the expression of MDK, VIM and TGFB1. The percentage of immuno-reactive cells was rated as follows: 0 points, <10%; 1 point, 10-50%; 2 points, >50%. The staining intensity was rated as follows: 0 (no staining or weak staining = light yellow), 1 (moderate staining = yellow brown) and 2 (strong staining = brown). The overall score for MDK/VIM/TGFB1 expression was the sum of points determined for the percentage of positively stained immuno-reactive cells and the expression, and an overall score ranging from 0 to 4 was assigned. For the statistical analysis, the patients were divided into a low expression group (an overall score between 0 and 2) and a high expression group (an overall score between 3 and 4) 30-32. The final score is the combination of independent scores assigned by the two pathologists, which was reported in this study. Any differences in the scores were resolved by discussion between the two pathologists.

## Results

### Single-cell expression atlas of cSCC ecosystems

**Figure 1:**
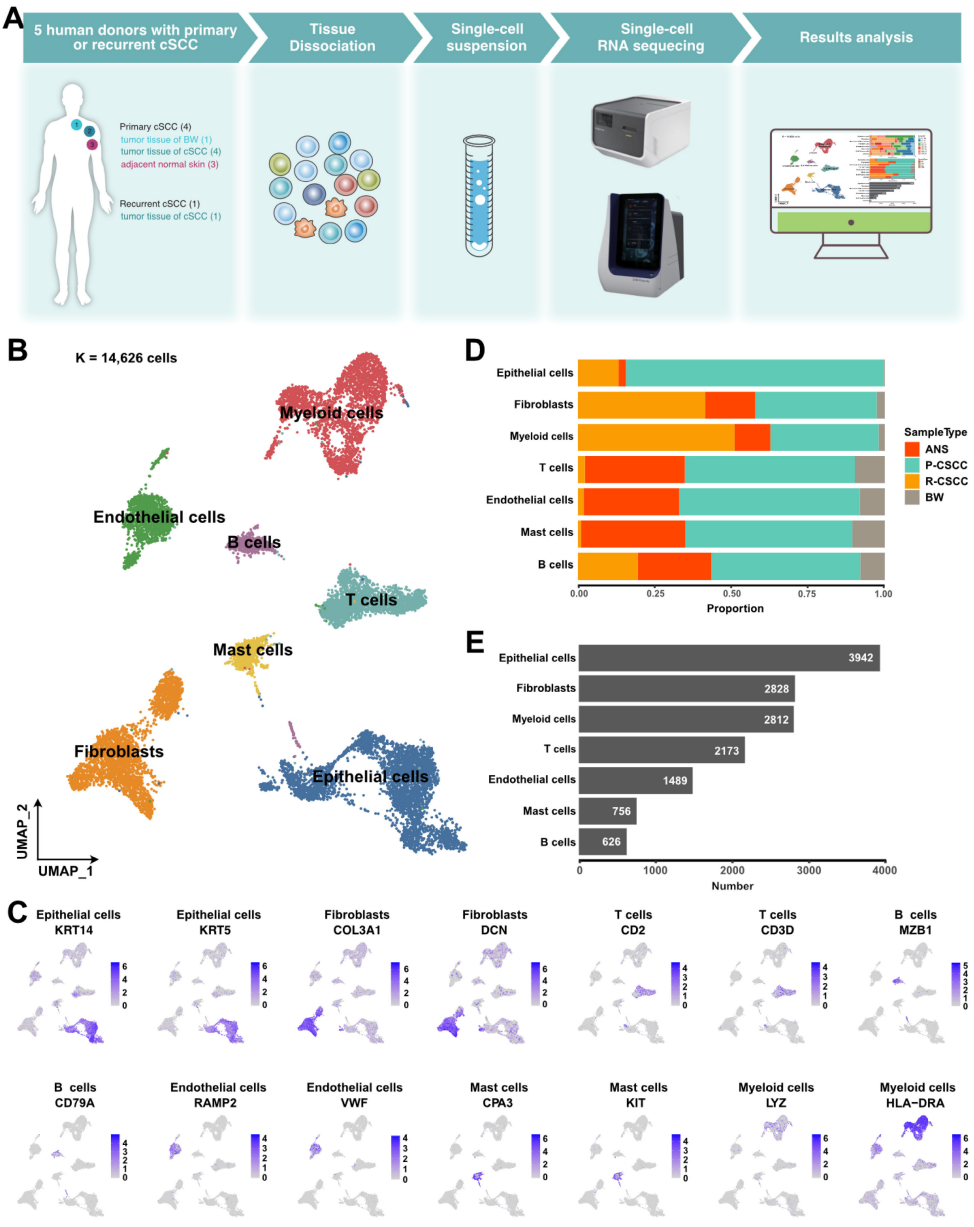
** A global overview of TME in cSCC. A.** Schematic graph describing the main workflow and study design. **B.** Uniform Manifold Approximation and Projection (UMAP) of major cell populations in our study, different colored dots represent different cell types. **C.** The expression level of cell type specific gene markers among UMAP. **D.** Sample type fractions relative to the total cell count per cell type.** E.** Bar plot shows the cell number of the major cells. ANS, adjacent normal skin. BW, Bowen disease. P-cSCC: primary cutaneous squamous cell carcinoma. R-cSCC: recurrent cutaneous squamous cell carcinoma.

The landscape and pathogenesis of TME were analyzed by performing scRNA-seq analysis of 6 tumor tissues and 3 adjacent normal skin tissues from patients diagnosed with primary or recurrent cSCC (Figure 1A). After quality control and several filtering steps, 14,626 high-quality cells were retained for scRNA-seq analysis (Figure S1A-B). All data were merged, and gene expression normalization, scaling, dimension reduction, batch correction, and cell clustering were performed to identify coarse cell types. Seven major cell types were detected based on the gene expression of canonical cell markers, including epithelial cells, fibroblasts, myeloid cells, T cells, endothelial cells, mast cells, and B cells (Figure 1B-C). The proportions of these major cell types varied greatly among different samples (Figure S1C), suggesting the heterogeneous character of the TME in cSCC. Primary tumors contained the highest cell abundance for most cell types, and recurrent cSCCs included a relatively high proportion of fibroblasts and B cells. In contrast, tumor tissue of Bowen disease (BW) comprised the lowest cell proportion (Figure 1D). Overall, epithelial cells, fibroblasts, and myeloid cells were the main components of the TME (Figure 1E).

### EMT characteristics of tumor-specific keratinocytes in recurrent cSCC

**Figure 2:**
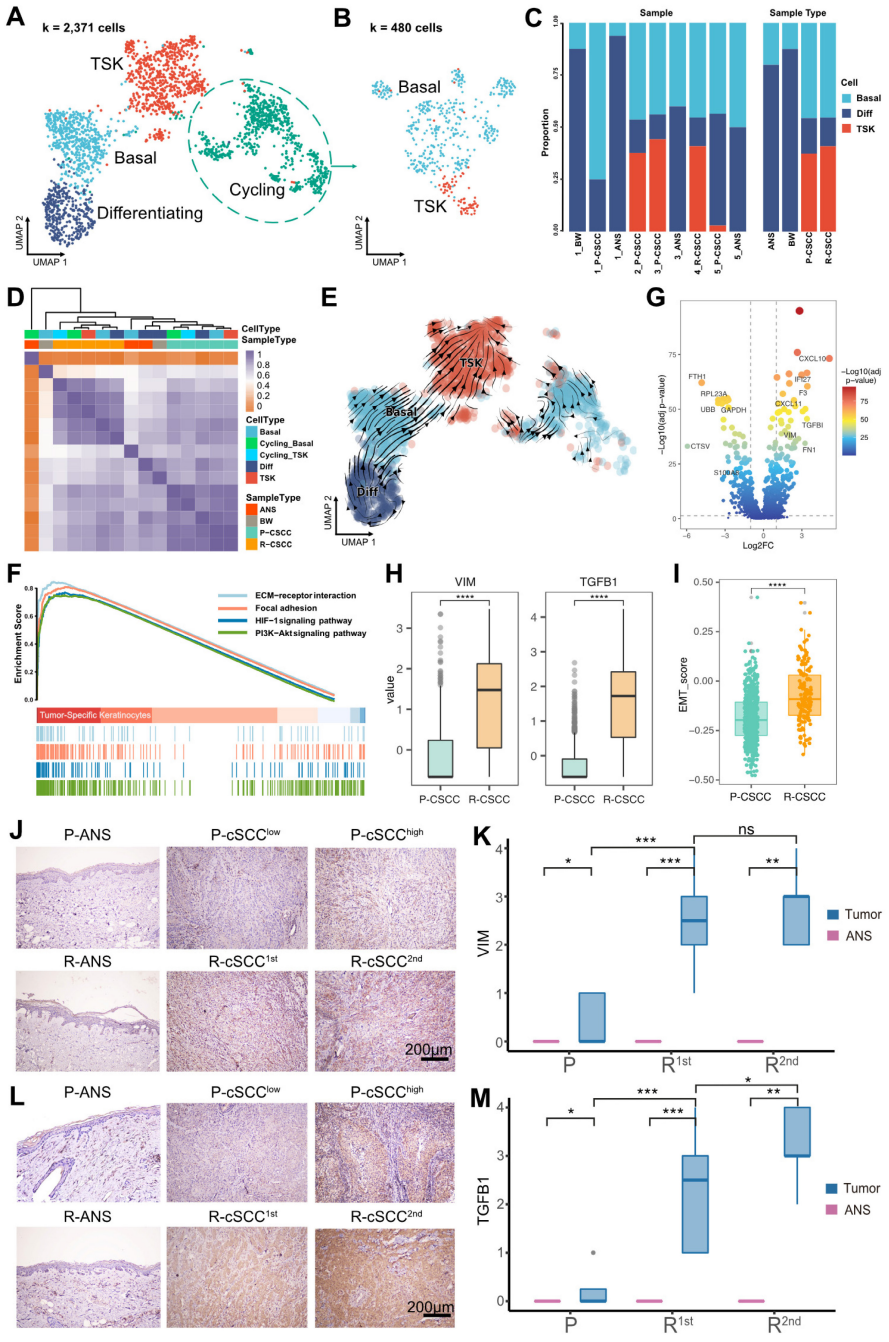
** Re-clustering and functional analysis of epithelial cells. A.** UMAP plot of sub-groups of epithelial cells.** B.** UMAP plot of cycling cells. **C.** The proportion of different cell types among samples and tissues. **D.** Heatmap depicting pairwise correlations between different cell types. **E.** Cell transition potential of basal, differentiating cells and TSKs determined by RNA velocity analysis.** F.** Enriched pathways of TSKs by GSEA analysis.** G.** Volcano plot showing the differential expressed genes between primary and recurrent cSCC.** H.** Gene expression level of VIM and TGFB1 across primary and recurrent cSCC. **I.** MET signature score of TSKs across primary and recurrent cSCC. **J.** The IHC staining of VIM in representative samples. **K.** The IHC score of VIM in primary, the first and second recurrence of cSCC, different tissue sites were marked by different color. **L.** The IHC staining of TGFB1 in representative samples. **M.** The IHC score of TGFB1 in primary, the first and second recurrence of cSCC, different tissue sites were marked by different color. Wilcoxon signed-rank test, ^****^p < 0.0001.

Overall, 3,942 epithelial cells were further reclassified into 4 clusters. We removed cell clusters that also expressed the gene markers of other cells, leaving 2,371 cells (Figure S2). Cells in each of the 4 clusters expressed known representative genes (Figure 2A, Figure S3A): (1) basal (COL17A1^+^), (2) cycling (MKI67^+^, TOP2A^+^), (3) differentiating (KRT1^+^), and (4) TSK (MMP10^+^, PTHLH^+^) cells 12.

Furthermore, the cycling cells were grouped into cycling TSKs and basal cells based on the expression of known markers (Figure 2B, Figure S3B). Overall, ANS and BW had a high proportion of differentiating cells, while primary and recurrent cSCCs had a relatively high proportion of TSKs (Figure 2C). Correlation analysis showed that epithelial cells from the same sample types were prone to cluster together (Figure 2D), demonstrating that epithelial cells are highly heterogeneous relative to sample site origin. RNA velocity analysis revealed that basal and differentiating cells tended to differentiate into TSKs (Figure 2E). Functional enrichment analysis showed that TSK cells were associated with PI3K-Akt, ECM-receptor interaction, focal adhesion, and HIF-1 signaling pathways (Figure 2F), demonstrated to be associated with tumor progression or resistance to cancer therapies 33-37.

We further evaluated the activity score of functional terms associated with “hallmarks of cancer”. TSK cells showed the highest score in most hallmarks, demonstrating their cancer characteristics, such as “Self Sufficiency in Growth Signals”, “Insensitivity to Antigrowth Signals”, and “Evading Apoptosis” (Figure S3C). We also identified differentially expressed genes in TSK cells between primary and recurrent cSCCs, and discovered that two EMT-related genes, VIM and TGFB1, were upregulated in recurrent cSCC (Figure 2G-H). Moreover, we calculated the EMT signature score of TSKs and found significantly higher EMT scores in recurrent cSCC (Figure 2I). We subsequently performed IHC staining to validate the expression of VIM and TGFB1 in cSCC in a clinical cohort that comprised 16 patients with cancer progression events, from primary tumors to cSCCs with single or multiple recurrences (Table S2). The results showed that both VIM and TGFB1 were significantly highly expressed in recurrent cSCCs (Figure 2J-M), implying that the recurrence of cSCC is associated with EMT.

### T cell-excluded microenvironment in recurrent cSCC

**Figure 3:**
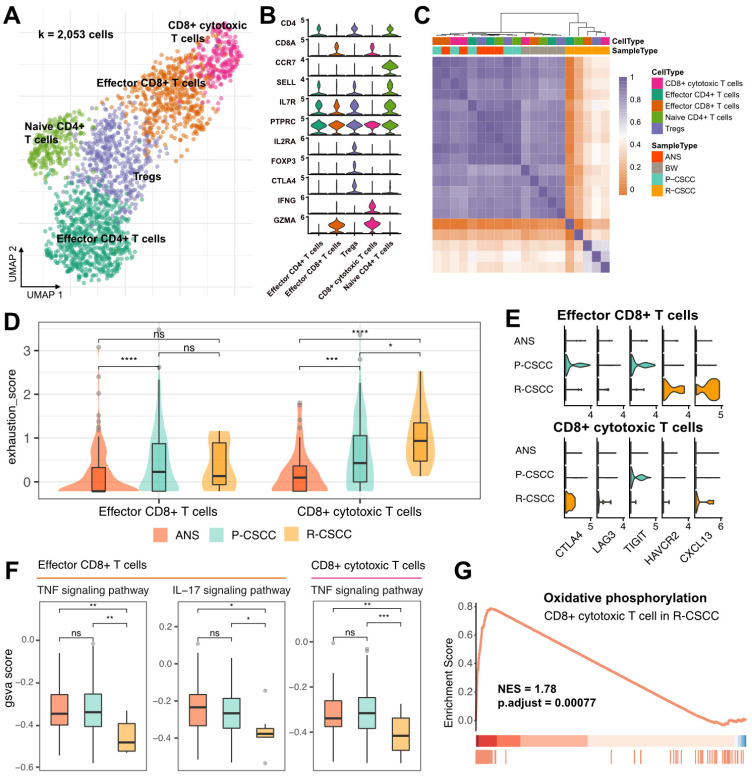
** T cell annotation and functional characterization. A.** UMAP visualization of T cells, different colors represent distinct sub-populations. **B.** Violin plot showing the representative markers of T cell lineage. **C.** Heatmap showing the pairwise correlations between T cells. **D.** The distribution of exhaustion score of effector CD8+ T cells and CD8+ cytotoxic T cells among ANS, primary and recurrent cSCC. **E.** The expression level of immune inhibitors among different sample and cell types. **F.** The GSVA score of inflammatory pathways in effector CD8+ T cells and CD8+ cytotoxic T cells among different sample types. **G.** Significantly identified pathway that enrich in recurrent versus primary cSCC. Wilcoxon signed-rank test, ^*^p < 0.05, ^**^p < 0.01, ^***^p < 0.001.

Subclustering of T cells identified five subpopulations: (1) effector CD4^+^ T cells (CD4^+^, CCR7^-^), (2) Tregs (CD4^+^, IL2RA^+^, FOXP3^+^, CTLA4^+^), (3) naive CD4^+^ T cells (CD4^+^, CCR7^+^, SELL^+^), (4) effector CD8^+^ T cells (CD8^+^, GZMA^+^), and (5) CD8^+^ cytotoxic cells (CD8^+^, IFNG^+^, GZMA^+^) (Figure 3A-B). RNA velocity analysis displayed a bidirectional flow between Tregs and effector CD4^+^ T cells and effector CD8^+^ T cells and CD8^+^ cytotoxic cells, while the differentiation from naive CD4^+^ T cells to Tregs appeared to be irreversible (Figure S4A). These findings indicated that Tregs, effector CD4^+^ T cells, effector CD8^+^ T cells, and CD8^+^ cytotoxic cells in the cSCC were prone to becoming intermediate and plastic and had the potential to differentiate into other cells.

Both CD4^+^ and CD8^+^ T cells were enriched in primary cSCC, while recurrent cSCC sites showed the lowest T cell infiltration (Figure S4B-C), indicating that recurrent cSCC tended to be a T cell desert tumor. The above-described T cells from different populations were further clustered based on their gene expression level. The results showed that T cells from ANS and primary cSCC tended to cluster together, and T cells from recurrent cSCC had a distinct pattern relative to other sites of origin (Figure 3C). Next, we evaluated the exhaustion score of effector CD8^+^ T cells and CD8^+^ cytotoxic T cells and compared it among ANS, primary and recurrent cSCCs. Compared with ANS, effector CD8^+^ T cells and CD8^+^ cytotoxic T cells showed increased exhaustion scores in primary cSCC. In contrast, CD8+ cytotoxic T cells had increased exhaustion scores in recurrent cSCC compared with ANS and primary cSCC (Figure 3D). Specifically, in primary cSCC, CTLA4 and T cell immunoreceptor with immunoglobulin and ITIM domain (TIGIT) displayed high expression levels in effector CD8^+^ T cells, and TIGIT showed high expression levels in CD8^+^ cytotoxic T cells. In recurrent cSCC, effector CD8^+^ T cells exhibited high expression levels of HAVCR2 (TIM3) and CXCL13, while CD8^+^ cytotoxic T cells displayed high expression levels of CTLA4 and CXCL13 and relatively higher expression levels of LAG3 (Figure 3E). This observation indicated that the exhaustion of CD8^+^ T cells in primary and recurrent cSCC was related to different inhibitors.

Furthermore, we evaluated an inflammatory score to gain deeper insight into the inflammatory changes among the ANS and primary and recurrent cSCCs, focusing on 4 pathways. For effector CD8^+^ T cells, primary cSCC exhibited activity scores similar to those of ANS, while recurrent cSCC showed significantly decreased inflammatory scores in the tumor necrosis factor (TNF) and IL-17 signaling pathways (Figure 3F, Figure S4D). For CD8^+^ cytotoxic T cells, except for the IL-17 signaling pathway, primary cSCC displayed an inflammatory score similar to that of ANS, while the activity scores for the TNF and NOD-like receptor signaling pathways showed significantly decreased levels in recurrent cSCC (Figure 3F, Figure S4E). In addition, GSEA showed upregulation of “oxidative phosphorylation” process in CD8^+^ cytotoxic T cells in recurrent cSCC compared to primary cSCC (Figure 3G, NES = 1.78, *p* < 0.01), demonstrating that cells were in a metabolically active state. Our analysis revealed that recurrent cSCC exhibited low infiltration and a decreased inflammatory score of T cells, which may be a key reason for cSCC recurrence.

### Protumor phenotypes of SPP1^+^ CD209^high/low^ TAMs in recurrent cSCC

**Figure 4:**
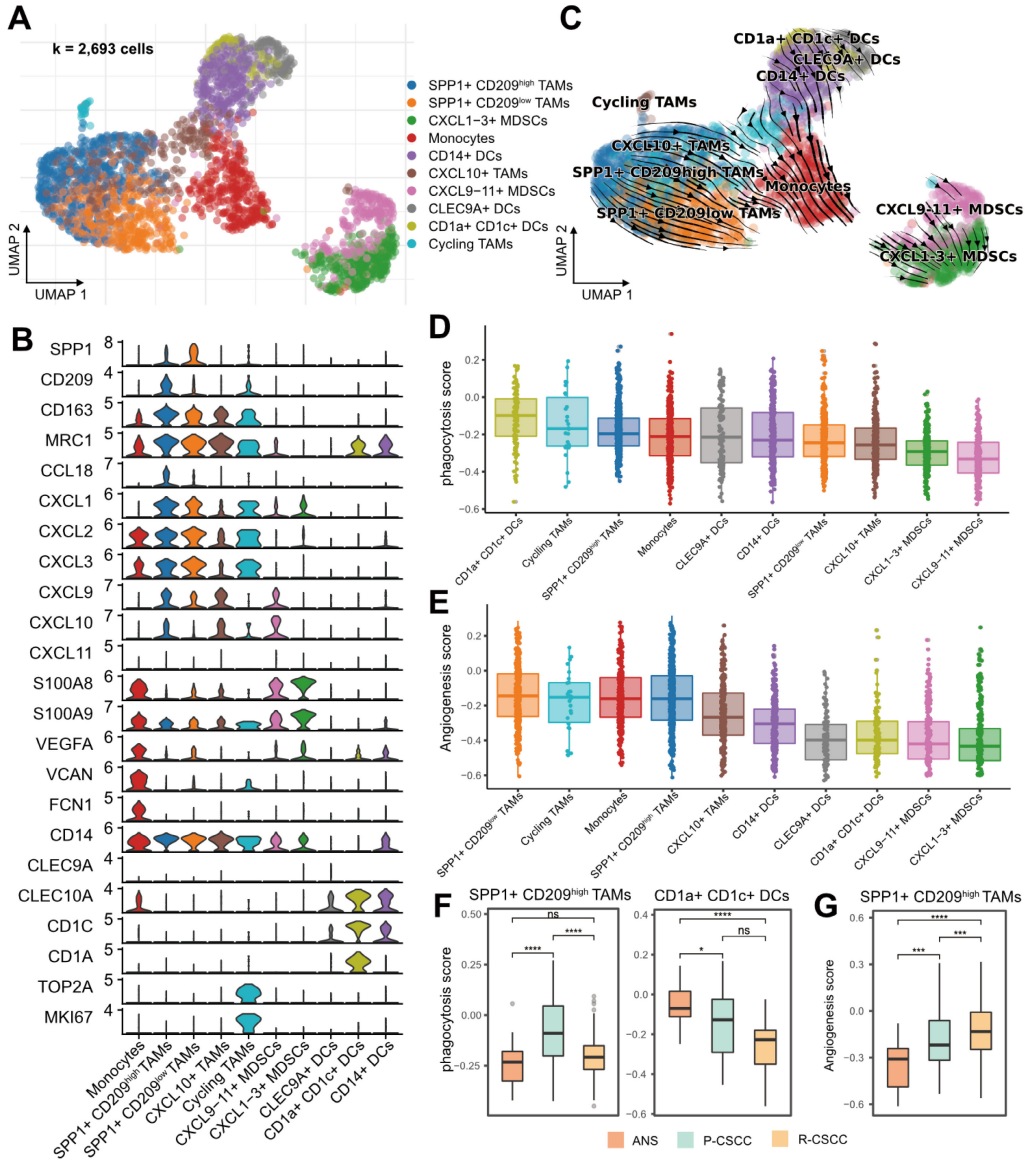
** Components and phenotypes of myeloid cells in cSCC. A.** UMAP plot of myeloid cells. **B.** Violin plot of the maker genes in myeloid sub-populations. **C.** UMAP plot showing the RNA velocity of myeloid cells. **D.** Distribution of phagocytosis score in each cell type, ranking by the median value. **E.** Distribution of angiogenesis in each cell type, ranking by the median value. **F.** The GSVA score of phagocytosis score in SPP1^+^ CD209^high^ TAMs and CD1a^+^ CD1c^+^ DCs among different sample types.** G.** The GSVA score of angiogenesis in SPP1^+^ CD209^low^ TAMs among different sample types. Wilcoxon signed-rank test, ^*^p < 0.05, ^***^p < 0.001, ^****^p < 0.0001.

Myeloid cells were among the most abundant cells in the TME of cSCC (Figure 1E) and have been demonstrated to participate in tumor progression and metastasis 38. Subsequently, 2,693 myeloid cells were clustered and annotated into 10 subpopulations (Figure 4A). In total, 4 populations were designated TAMs, which had various features: (1) SPP1^+^ CD209^high^ TAMs (SPP1^+^, CD209^high^, CD163^+^, MRC1^+^, CCL18^+^), (2) SPP1^+^ CD209^low^ TAMs (SPP1^+^, CD209^low^, CD163^+^, MRC1^+^, CCL18^+^), (3) CXCL10^+^ TAMs (CD163^+^, MRC1^+^, CXCL10^+^), and (4) cycling TAMs (CD209^+^, CD163^+^, MRC1^+^, TOP2A^+^, MKI67^+^). In addition, one cluster was identified as monocytes (VEGFA^+^, VCAN^+^, FCN1^+^), and 3 subpopulations were characterized as DCs: (1) CD14^+^ DCs (CD1A^-^, CD14^+^, CD1C^+^), (2) CLEC9A^+^ DCs (CLEC9A^+^, CD1C^+^), and (3) CD1a^+^ CD1c^+^ DCs (CLEC10A^+^, CD1A^+^, CD1C^+^, CD14^-^). We divided MDSCs into 2 subgroups: (1) CXCL9-11^+^ MDSCs (CXCL9^+^, CXCL10^+^, CXCL11^+^, IL1B^+^, S100A8^+^, S100A9^+^) and (2) CXCL1-3^+^ MDSCs (CXCL1^+^, CXCL2^+^, CXCL3^+^, IL1B^+^, S100A8^+^, S100A9^+^). The expression of each cell-specific gene marker was presented in Figure 4B and S5. Notably, for the two groups of MDSCs identified in our study, CXCL9-11^+^ MDSCs were enriched in recurrent cSCC, while CXCL1-3^+^ MDSCs were more abundant in primary cSCC (Figure S6A-B). Compared with CXCL1-3^+^ MDSCs, CXCL9-11^+^ MDSCs were enriched in the pathways of “oxidative phosphorylation” and “antigen processing and presentation” when analyzed by GSEA (Figure S6C).

RNA velocity analysis showed the transition directions from TAMs and DCs to monocytes, and MDSCs were located at the end of the differentiation trajectory (Figure 4C). Furthermore, using CytoTRACE, we confirmed that the potential origins of monocytes were M2 macrophages and DCs (Figure S6D) 29. Recent studies reported that *Bordetella pertussis* adenylate cyclase toxin could inhibit the differentiation of infiltrating monocytes into macrophages and DCs by activating cMAP signaling and could provoke the dedifferentiation of macrophages to monocyte-like cells 39. Therefore, we examined the cMAP activity score of myeloid cells over time. Interestingly, there was a significantly negative correlation between latent time and cMAP score (R = 0.32, *p* < 0.00001). The cMAP score displayed a decreased pattern when macrophages and DCs transformed into monocytes, and no significant reduction pattern in the cMAP score was observed for cells originating from monocytes (Figure S6E-F). These observations suggested that the transmission of monocytes from macrophages and DCs might be related to the cMAP signaling pathway, and the transmission direction might be related to the malignancy level of cSCC.

Recently, a subtype of TAMs with SPP1^+^ characteristics and angiogenesis-related properties was reported to be associated with tumor metastasis 13, 40, 41. In our study, SPP1^+^ CD209^high^ and SPP1^+^ CD209^low^ TAMs were identified as two subpopulations of SPP1^+^ TAMs with a high proportion in recurrent cSCC (Figure S6A-B). We examined the angiogenic and phagocytotic properties of TAMs and found different characteristics in these subpopulations. SPP1^+^ CD209^high^ TAMs exhibited a higher phagocytosis score than SPP1^+^ CD209^low^ TAMs, while SPP1^+^ CD209^low^ TAMs had the highest angiogenesis score (Figure 4D-E). In addition, CD1a^+^ CD1c^+^ DCs displayed the highest phagocytosis score (Figure 4D). Not surprisingly, the phagocytosis score of SPP1^+^ CD209^high^ TAMs, CD1a^+^ CD1c^+^ DCs, and cycling TAMs decreased in recurrent cSCC compared with primary cSCC (Figure 4F, Figure S6G). Also, SPP1^+^ CD209^high^ TAMs displayed significantly increased angiogenesis scores in primary and recurrent cSCCs (Figure 4G, Figure S6H).

Next, we evaluated the inflammatory pathway scores of these myeloid cells. Overall, TAMs and monocytes tended to have higher inflammatory scores (Figure S7A-D). For the TNF, NF-kappa B, and NOD-like receptor signaling pathways, SPP1^+^ CD209^high^ TAMs had elevated scores in primary cSCC and decreased scores in recurrent cSCC (Figure S7E, G-H). For the NF-kappa B and IL-17 signaling pathways, monocytes showed lower scores in primary and recurrent cSCC than in ANS (Figure S7E-F). Collectively, our results revealed that SPP1^+^ TAMs had lower phagocytosis and inflammation but higher angiogenesis scores in recurrent cSCC, potentially reflecting the cSCC recurrence mechanism.

### Crosstalk between recurrent cSCC-enriched IL7R^+^ CAFs and tumor-specific keratinocytes

**Figure 5:**
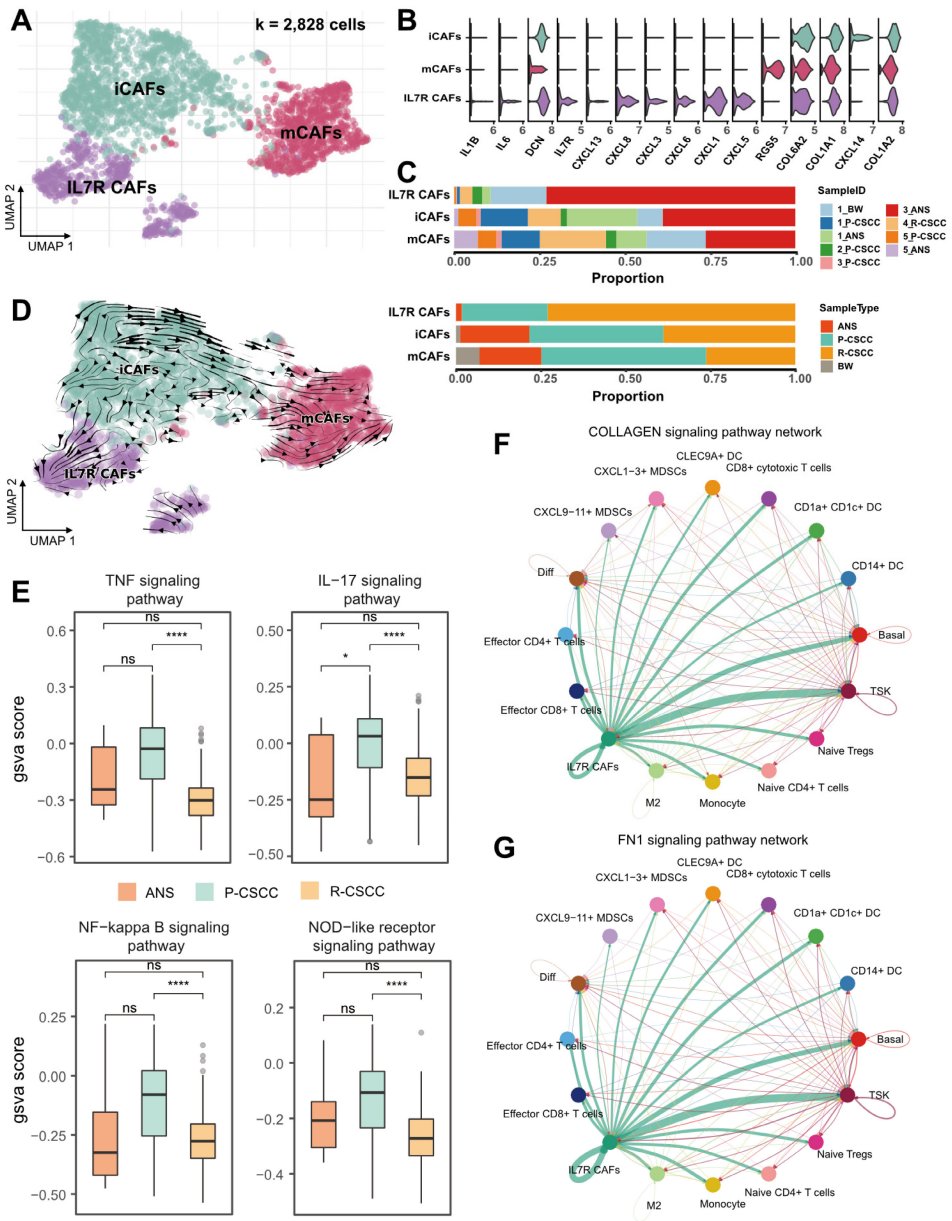
** Assessing the functional states of fibroblasts in cSCC. A.** UMAP plot showing the sub-populations of fibroblasts. **B.** Violin plot showing the high expression of gene markers in fibroblasts. **C.** The proportion of fibroblasts relative to sample ID and sample type. **D.** RNA velocity plot showing cell transition directions among fibroblasts. **E.** The GSVA score of inflammatory pathways in fibroblasts among different sample types. **F.** Cell-cell interactions in COLLAGEN signaling pathway. **G.** Cell-cell interactions in FN1 signaling pathway. Wilcoxon signed-rank test, ^****^p < 0.0001.

In our study, 2,828 fibroblasts were classified into 3 subpopulations based on the expression of marker genes, including (1) mCAFs (RGS5^+^, DCN^+^, COL6A2^+^, COL1A1^+^, COL1A2^+^), (2) iCAFs (RGS5^-^, DCN^+^, COL6A2^+^, COL1A1^+^, COL1A2^+^), and (3) IL7R**^+^** CAFs (IL7R^+^, IL1B^+^, IL6^+^, CXCL1^+^, CXCL3^+^, CXCL5^+^, CXCL6^+^, CXCL8^+^, CXCL13^+^, CXCL14^+^, DCN^+^, COL6A2^+^, COL1A1^+^, COL1A2^+^) (Figure 5A-B). Notably, IL7R**^+^** CAFs were more abundant in recurrent cSCC, while primary cSCC had a relatively high proportion of mCAFs (Figure 5C). RNA velocity analysis revealed the transition direction from iCAFs to mCAFs and iCAFs to IL7R**^+^** CAFs (Figure 5D), indicating that iCAFs could differentiate into mCAFs and IL7R**^+^** CAFs, which might be related to primary and recurrent cSCC, respectively.

Next, we calculated the inflammatory score of IL7R**^+^** CAFs and compared it between ANS, primary, and recurrent cSCCs. We found that in all four investigated pathways, primary cSCC showed a slight increase in the inflammatory score, which was significantly decreased in recurrent cSCC (Figure 5E), indicating a low inflammatory feature for IL7R**^+^** CAFs during cSCC recurrence. We performed cell-cell communication analysis using CellChat to gain a deeper insight into the biological function of IL7R**^+^** CAFs 21. Interestingly, IL7R**^+^** CAFs expressed high levels of ligands related to EMT, such as collagens [encoded by COL1A1, COL1A2, COL4A1, COL6A1, COL6A2, COL6A3], fibronectin 1 [encoded by FN1], tenascin C [encoded by TNC], and Thy-1 [encoded by THY1] (https://www.gsea-msigdb.org/gsea/msigdb/cards/HALLMARK_EPITHELIAL_MESENCHYMAL_TRANSITION.html). The receptors for these ligands were expressed by a wide range of cells, which subsequently induced typical cell-cell interactions (Figure 5F-G, Figure S8A-F). Notably, IL7R**^+^** CAFs showed the strongest interaction with TSKs in the collagen, FN1, and TEAD signaling pathways.

For the receptors that are expressed in TSKs, integrins [encoded by ITGA1, ITGA2, ITGA3, ITGA4, ITGA5, ITGA8, ITGAV, ITGAX, ITGAM, ITGB1, and ITGB2] can recognize multiple ligands, including collagens, fibronectin 1, tenascin C, and Thy-1, causing downstream crosstalk between TSKs and IL7R**^+^** CAFs. Integrin signaling is known to have a profound effect on tumor cells, including proliferation, migration, and survival 42. Also, ITGA3 has been shown to promote endothelial cell motility and angiogenesis during the early stages of neovascularization 43. Similarly, ITGA8 has been demonstrated to regulate the recruitment of mesenchymal cells into epithelial structures and promote cell survival 44, 45. Therefore, IL7R**^+^** CAFs might promote the EMT of TSKs by cell-cell communication via multiple signaling pathways in recurrent cSCC.

### Role of the MDK-dependent pathway in recurrent cSCC by cell-cell interaction analysis

**Figure 6:**
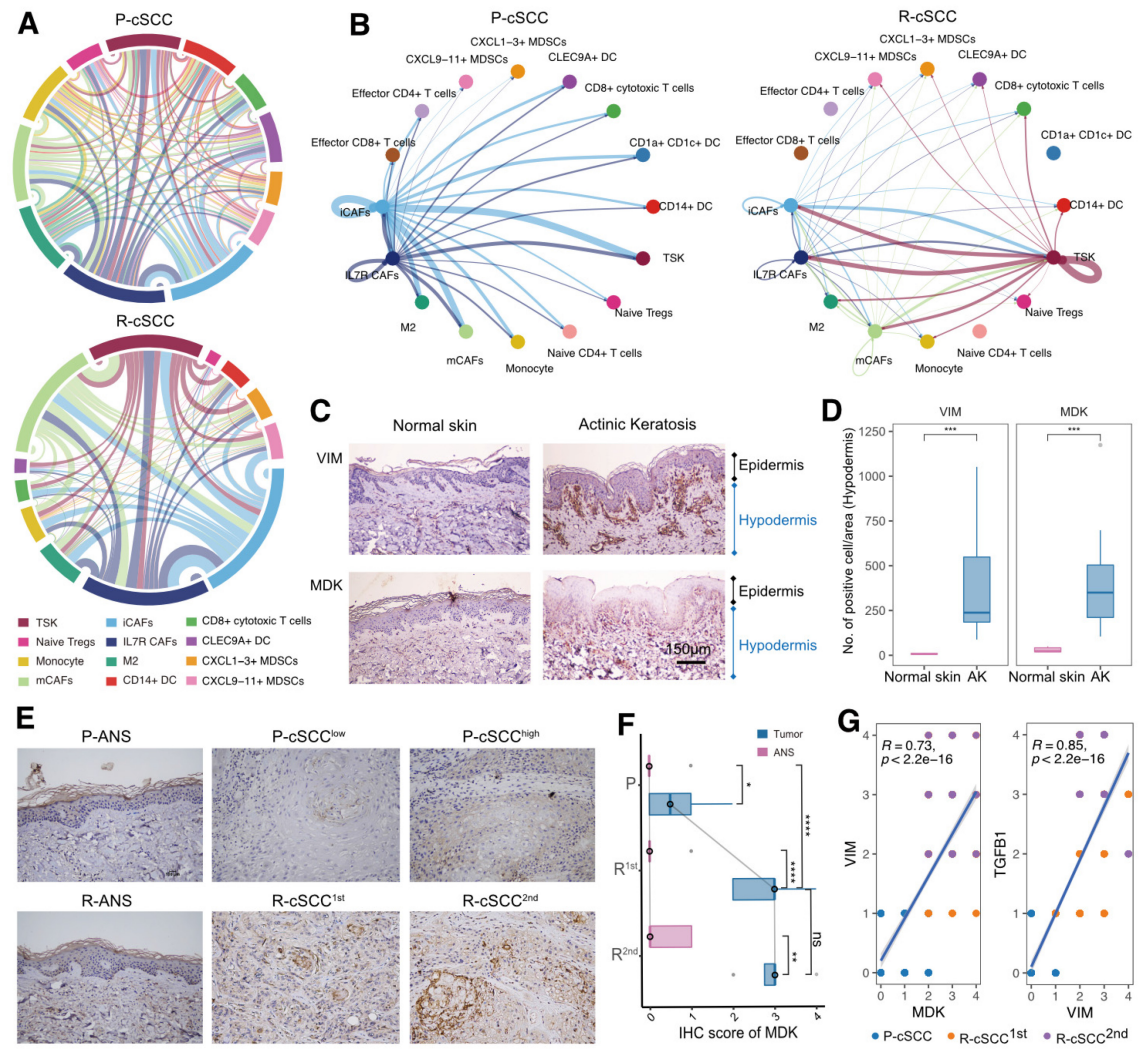
** Cell-cell interactions of the primary and recurrent cSCC. A.** Dependency wheel illustrating the interaction relationship between cells within the TME of primary and recurrent cSCC, the link size represents the interaction number. **B.** Cell-cell interactions of MDK signaling pathway in primary and recurrent cSCC, the link size represents the interaction strengthen. **C.** The IHC staining of VIM and MDK in representative samples. **D.** VIM and TGFB1 in hypodermis (number of positive cell/area), different tissue sites were marked by different color.** E.** The IHC staining of MDK in representative samples. **F.** The IHC score of MDK in primary, the first and second recurrence of cSCC, different tissue sites were marked by different color. **G.** Scatter plot of the score of MDK and VIM, MDK and TGFB1 in tumor samples of cSCC. Different dots represent the type of sample. Wilcoxon signed-rank test, ^*^p < 0.05,^ **^p < 0.01, ^****^p < 0.0001.

Previous evidence suggested differences in the composition and functional status of the TME between primary and recurrent cSCC. We hypothesized that primary and recurrent cSCC display distinct subcellular interaction relationships within the TME, which may profoundly influence the tumor phenotype. We tested this hypothesis by performing cell-cell communication analysis separately in primary and recurrent cSCCs. The results showed that primary cSCC had many more cell interaction events than recurrent cSCC (6696 vs. 1196), which could have been due to the small cell number in recurrent cSCC (8217 vs. 3341). Notably, iCAFs and IL7R**^+^** CAFs had more interactions with other cells in primary cSCC. In contrast, TSKs, iCAFs, mCAFs, and IL7R**^+^** CAFs were dominant cells that communicated with other cells in recurrent cSCC (Figure 6A). In addition, we calculated the incoming and outgoing interaction strengths of cells and found that TSKs played an essential role within the TME in recurrent cSCC compared with primary cSCC (Figure S9A).

We subsequently focused on TSKs and identified increased interaction signaling in recurrent cSCC using primary cSCC as a control. Among the significantly upregulated pathways, the MDK pathway was exclusively present in recurrent cSCC (Figure S9B). MDK is a heparin-binding growth factor and has been reported to serve as an important regulator that supports cell transformation, growth, survival, migration, and angiogenesis in several human cancers 46, 47. Interestingly, primary and recurrent cSCCs possessed distinct MDK-associated interaction relationships. In primary cSCC, iCAFs and IL7R**^+^** CAFs produced high levels of MDK to mediate their intercellular communications, while in recurrent cSCC, TSKs secreted high levels of MDK, whose receptors were expressed on TSKs and other cells, thus regulating the strong cell-cell interactions within TSKs (Figure 6B, Figure S9C, Figure S10).

We examined the effect of MDK on fibroblasts by investigating the expression of MDK and VIM (a general marker of fibroblasts) in normal skin and actinic keratosis (AK) samples. We also validated the expression of MDK by IHC staining using 5 normal skin samples, 10 samples with AK, and 16 patients with primary tumors and single or multiple recurrences of cSCC, comprising matched ANS and tumor samples (Table S2, S3). Compared with normal skin, VIM was upregulated in the hypodermis of AK, indicating the presence of increased fibroblasts in the hypodermis (Figure 6C-D). MDK was also highly expressed in the hypodermis of AK and displayed a positive correlation with VIM (R = 0.58, *p* = 0.088, Figure S11A). Therefore, we hypothesized that cSCC had increased fibroblasts in early carcinogenesis mediated by MDK. Surprisingly, MDK also participated in the recurrence of cSCC, and tumor tissues with the first and second recurrences had higher MDK expression levels than primary cSCC (Figure 6E-F). Besides, MDK was significantly correlated with the expression of VIM and TGFB1 (Figure S11B), indicating that it may regulate the EMT of cSCC. MDK was reported to be abnormally expressed in human malignancies and participate in diverse cancer development and progression processes 46, suggesting that MDK expressed on different cells may be a double-edged sword in the TME. When fibroblasts highly express MDK, it may promote fibroblast replication and block the proliferation and metastasis of tumor cells through cellular interactions, thus avoiding tumor recurrence. Conversely, TSKs may acquire proliferative or EMT capacity in recurrent tumor tissues by expressing MDK, which then mediates tumor recurrence.

**Figure 7:**
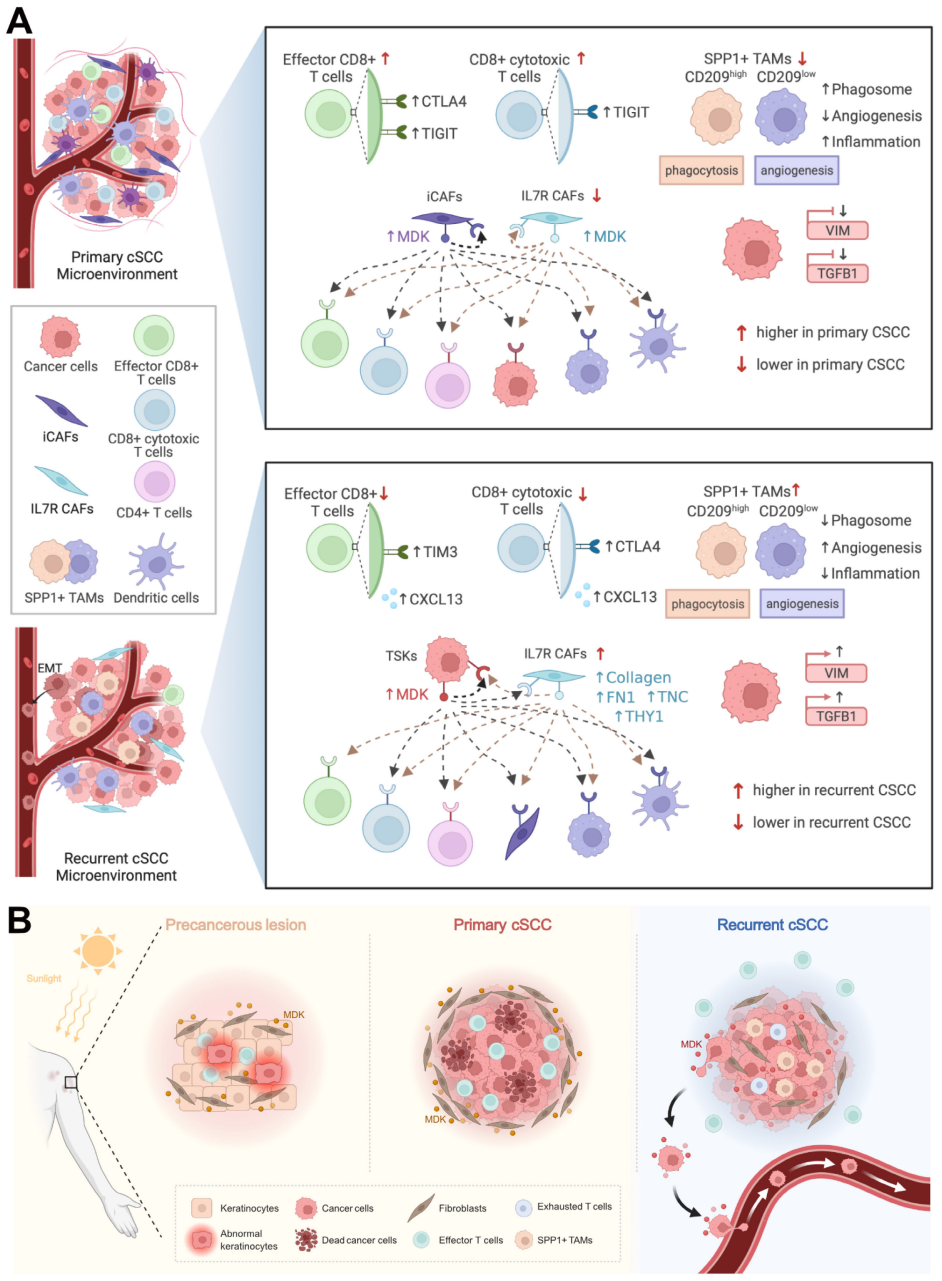
**Diagram illustrating the reprogramming of TME in cSCC progression. A.** Top, schematic diagram of the TME and cell-cell interactions in primary cSCC. Bottom, schematic diagram of the TME and cell-cell interactions in recurrent cSCC. **B.** The specific transformation pattern of MDK within TME, ranging from a precursor AK, to primary cSCC, and finally recurrent cSCC.

Taken together, we found that primary cSCC was similar to a “hot” tumor, which infiltrated with a higher abundance of T cells, whereas recurrent cSCC tended to be a “cold” tumor and harbored EMT characteristics. CD8^+^ T cells secreted high levels of CTLA4 and TIGIT in primary cSCC, while they highly expressed TIM3, CTLA4, and CXCL13 in recurrent cSCC. Relatively high proportions of SPP1^+^ CD209^high^ and SPP1^+^ CD209^low^ TAMs, characterized by marked features of phagocytosis and angiogenesis, respectively, were observed in recurrent cSCC with low potential for phagocytosis and inflammation and obvious angiogenic characteristics in recurrent cSCC. We observed that MDK could drive strong intercellular interactions within iCAFs and IL7R**^+^** CAFs in primary cSCC, while TSKs tended to have strong interactions with themselves by secreting MDK. Also, in recurrent cSCC, IL7R**^+^** CAFs showed interactions with TSKs by expressing EMT-related markers, possibly promoting the EMT of tumor cells (Figure 7A).

## Discussion

Although cSCC is usually not life-threatening, and can mostly be successfully eradicated by surgical resection, a subset of cSCC can recur and metastasize, leading to death 48. For cancer cell progression and metastasis, a highly complex and heterogeneous microenvironment is essential 49. Here, we performed scRNA-seq on 14,626 single cells of ANS and tumor tissues from 5 patients diagnosed with primary or recurrent cSCC. Through integrated analyses of scRNA-seq data, we provided a comprehensive functional and cell-cell interaction landscape of epithelial cells, fibroblasts, myeloid cells, and T cells, which are the major components of the cSCC TME. Our study characterized the heterogenous and functional differences in the TME between primary and recurrent cSCC. The critical reprogramming of the TME described in our study revealed the mechanism of cSCC recurrence and may provide instructive guidance for clinical prevention and treatment.

We found that recurrent cSCC was characterized by low T-cell infiltration and elevated T-cell exhaustion. Tumor-infiltrating CD8^+^ T cells have been shown to progress to an exhaustion state in many tumors 50, such as hepatocellular carcinoma 51, melanoma 52, and breast cancer 53. High expression of immune checkpoint receptors is associated with the exhaustion state of CD8^+^ T cells, such as PD-1, CTLA-4, TIM-3, and LAG-3 54, and their dysfunctional state has been demonstrated to affect the postoperative survival and recurrence risk of patients 51. Tumors characterized by a lack of effector T cells have been termed “cold tumors” and shown to resist immunotherapy 55, indicating that this treatment modality is inappropriate for recurrent cSCC. Inflammatory pathways play important roles in regulating the innate and adaptive immune response. TNF and NF-κB signaling can promote the activation of effector T cells 56, 57. IL-17 is a proinflammatory cytokine secreted by T cells that plays a critical role in host defense against bacterial infection 58. The NOD-like receptor signaling pathway participates in the innate immune response regulation of the host 59. In particular, T cells in recurrent cSCC exhibited decreased activity of the TNF, IL-17, NF-kappa B, and NOD-like receptor signaling pathways, which may explain their low defense and weak killing ability.

A recent study described that SPP1^+^ macrophages might prevent the infiltration of lymphocytes, further reducing the efficacy of PD-L1 treatment 40. Moreover, SPP1^+^ macrophages were reported to carry out angiogenesis positively correlated with EMT markers and related to tumor metastasis 13, 40, 41, 60. In our study, SPP1^+^ TAMs expressed a global protumor characteristic in recurrent cSCC, including lower phagocytosis and inflammation scores and higher angiogenesis scores, which may be the critical mechanism of cSCC recurrence. However, the two subpopulations of SPP1^+^ macrophages (SPP1^+^ CD209^high^ and SPP1^+^ CD209^low^) possessed different characteristics, showing remarkable features of phagocytosis and angiogenesis, respectively. This observation indicated the heterogeneous property of SPP1^+^ TAMs, implying that the proportion of SPP1^+^ CD209^high^ and SPP1^+^ CD209^low^ TAMs may affect the outcomes of cSCC. Patients with high proportions of SPP1^+^ CD209^high^ TAMs with strong phagocytosis ability might have a good prognosis. We found that recurrent cSCC had significantly higher expression levels of two EMT-associated genes, VIM and TGFB1, and high EMT characteristics, which may be related to increased numbers of SPP1^+^ TAMs.

The high expression of VIM and TGFB1 in recurrent cSCC was further validated using IHC in an independent clinical cohort. The TME is a heterogeneous collection of multiple cells and noncellular tissue components. Among them, CAFs act as a double-edged sword with tumor-restraining/promoting roles, which could induce EMT in diverse cancers 61-63. Here, we found that IL7R**^+^** CAFs were enriched in recurrent cSCC and, by expressing integrins, could interact with TSKs through the collagen, FN1, tenascin, and THY1 signaling pathways. Collagen and tenascin C (TNC) can promote EMT in tumors 64, 65; therefore, the interaction between TSKs and IL7R**^+^** CAFs may mediate the EMT of tumor cells in recurrent cSCC.

MDK is a heparin-binding growth factor that promotes the proliferation and EMT of tumor cells 46 and has been shown to correlate with tumor progression and poor prognosis in glioblastoma 66. It could also lead to immunotherapy resistance and promote immunosuppression in human cancers 67. AK is the most common form of precancerous lesion in cSCC, which is related to cumulative ultraviolet (UV) exposure from sunlight 68. We measured VIM abundance, a general marker of fibroblasts, in normal skin and AK and found that it was significantly enriched in the hypodermis of AK. In addition, MDK was upregulated and tended to have an expression pattern consistent with VIM in AK. When fibroblasts were isolated from primary cSCC and healthy dermis, those derived from cSCC had increased proliferation compared to normal fibroblasts 69. In our study, the high expression of MDK in fibroblasts led to strong intercellular communication in primary cSCC. This evidence indicated that MDK might drive the massive proliferation of fibroblasts in AK under cumulative exposure to sunlight, which could be a protective mechanism against UV. Thus, when AK progresses to primary cSCC, MDK may further promote fibroblast proliferation, allowing it to regulate ECM remodeling and protect tumor cells from escaping the microenvironment. This hypothesis explains the clinical observation that primary cSCC is unlikely to metastasize. In recurrent cSCC, MDK was expressed at low levels in fibroblasts and shifted to a high expression pattern in cSCC, further promoting cell-cell communication within themselves. Besides, MDK was positively correlated with VIM and TGFB1 in a cSCC cohort, demonstrating that it is associated with EMT. Thus, MDK potentially drives the proliferation and EMT of tumor cells in recurrent cSCC. Our study proposes a specific transformation pattern of MDK, ranging from a precursor AK to primary cSCC and finally recurrent cSCC, providing a novel potential treatment target in cSCC (Figure 7B).

## Supplementary Material

Supplementary figures and tables.

## Abbreviations

cSCC: cutaneous squamous cell carcinoma
TME: tumour microenvironment
scRNA-seq: single-cell RNA sequencing
TAM: tumour-associated macrophage
TSK: tumour-specific keratinocyte
CAF: cancer-associated fibroblast
EMT: epithelial-mesenchymal transition
BCC: basal cell carcinoma
BW: Bowen disease
ANS: adjacent normal skin
UMI: unique molecular identifier
RSEC: recursive substitution error correction
PCA: principal component analysis
UMAP: Uniform Manifold Approximation and Projection
DEG: differentially expressed gene
KEGG: Kyoto Encyclopedia of Genes and Genomes
GSEA: gene set enrichment analysis
