# Supplementary material for: Signatures of EMT, immunosuppression, and inflammation in primary and recurrent human cutaneous squamous cell carcinoma at single-cell resolution
Source: Theranostics. 2022 Oct 31;12(17):7532–49. doi: 10.7150/thno.77528 (PMC9691356; doi:10.7150/thno.77528)
Supplement: Supplementary file 1 — Supplementary figures and tables. [file thnov12p7532s1.pdf]

Supplemental Material

Signatures of EMT, immunosuppression, and inflammation in primary and recurrent human cutaneous squamous cell carcinoma at single-cell resolution

Xin Li[#], Shuang Zhao[#], Xiaohui Bian[#], Lining Zhang, Lixia Lu, Shiyao Pei, Liang Dong, Wensheng Shi, Lingjuan Huang, Xiyuan Zhang, Mingliang Chen, Xiang Chen[*], Mingzhu Yin[*]

This file includes:

Figures S1-S10

Tables S1-S3

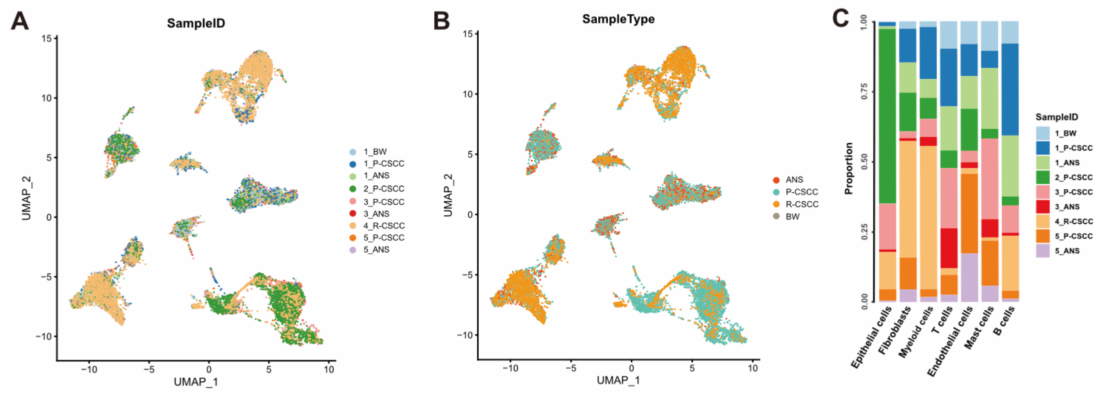

Figure S1. Visualization of single cells profiled in our study. A. UMAP plot colored by samples. **B.** UMAP plot colored by sample types. **C.** Sample fractions relative to the total cell count per cell type.

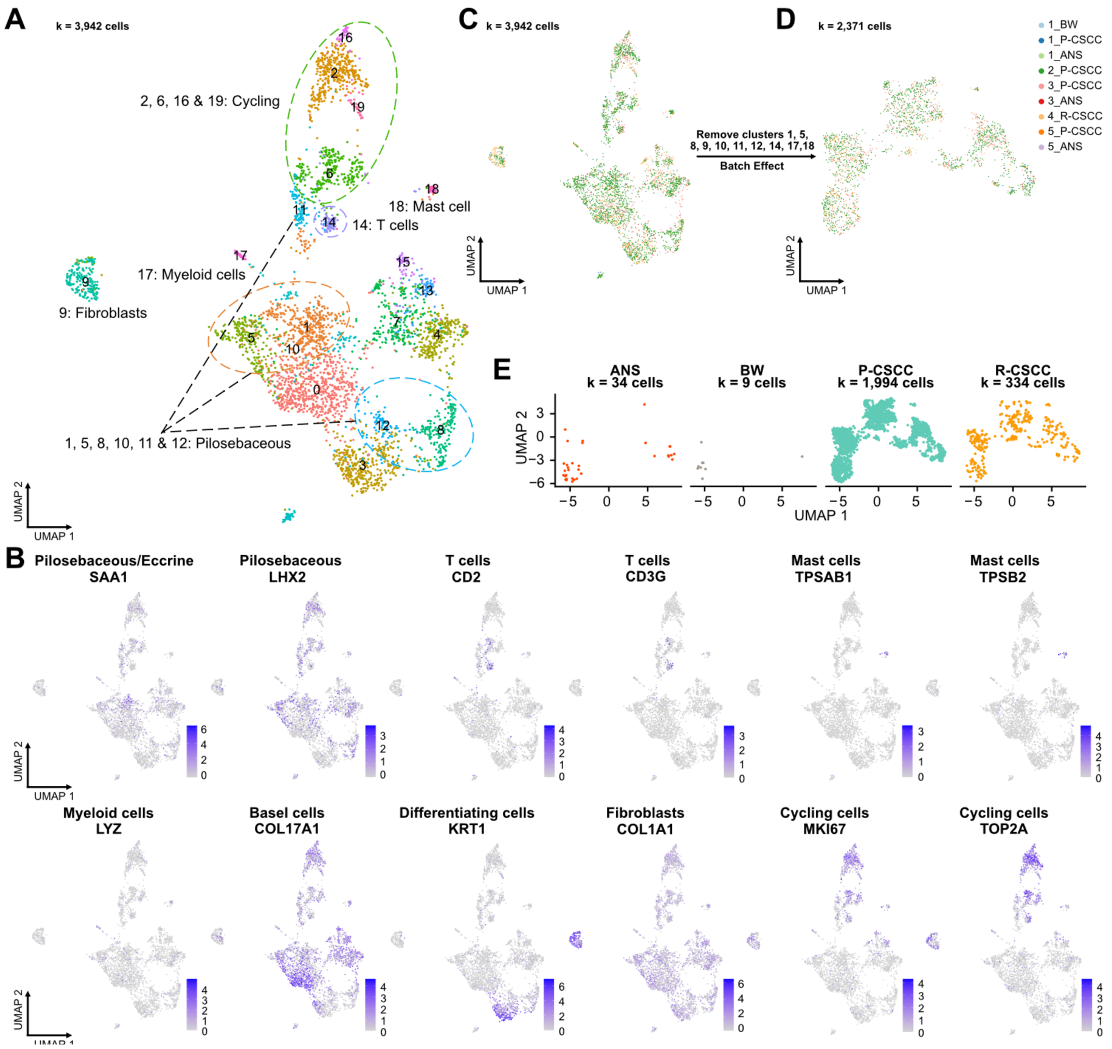

Figure S2. Epithelial cells clustering and annotation. A. Initial classification results of epithelial cells. **B.** The expression of specific cell markers among UMAP. **C.** UMAP plot colored by samples before removing doublets. **D.** UMAP plot colored by samples after removing doublets, re-scaling, and clustering. **E.** UMAP plot colored by sample types after removing doublets, re-scaling, and clustering.

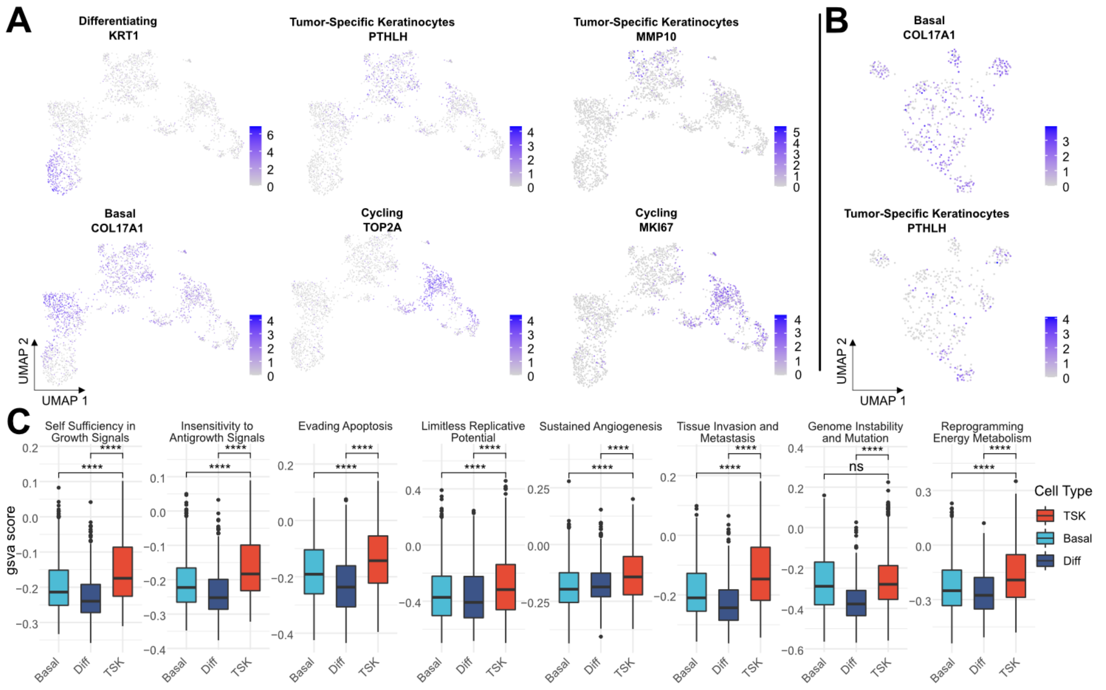

Figure S3. Functional characterization of epithelial cells in cSCC. A-B UMAP plot shown the expression level of epithelial cell associated markers. **C** The GSVA scores of "hallmarks of cancer" among different sample types. Wilcoxon signed-rank test, $^{****}p < 0.0001$.

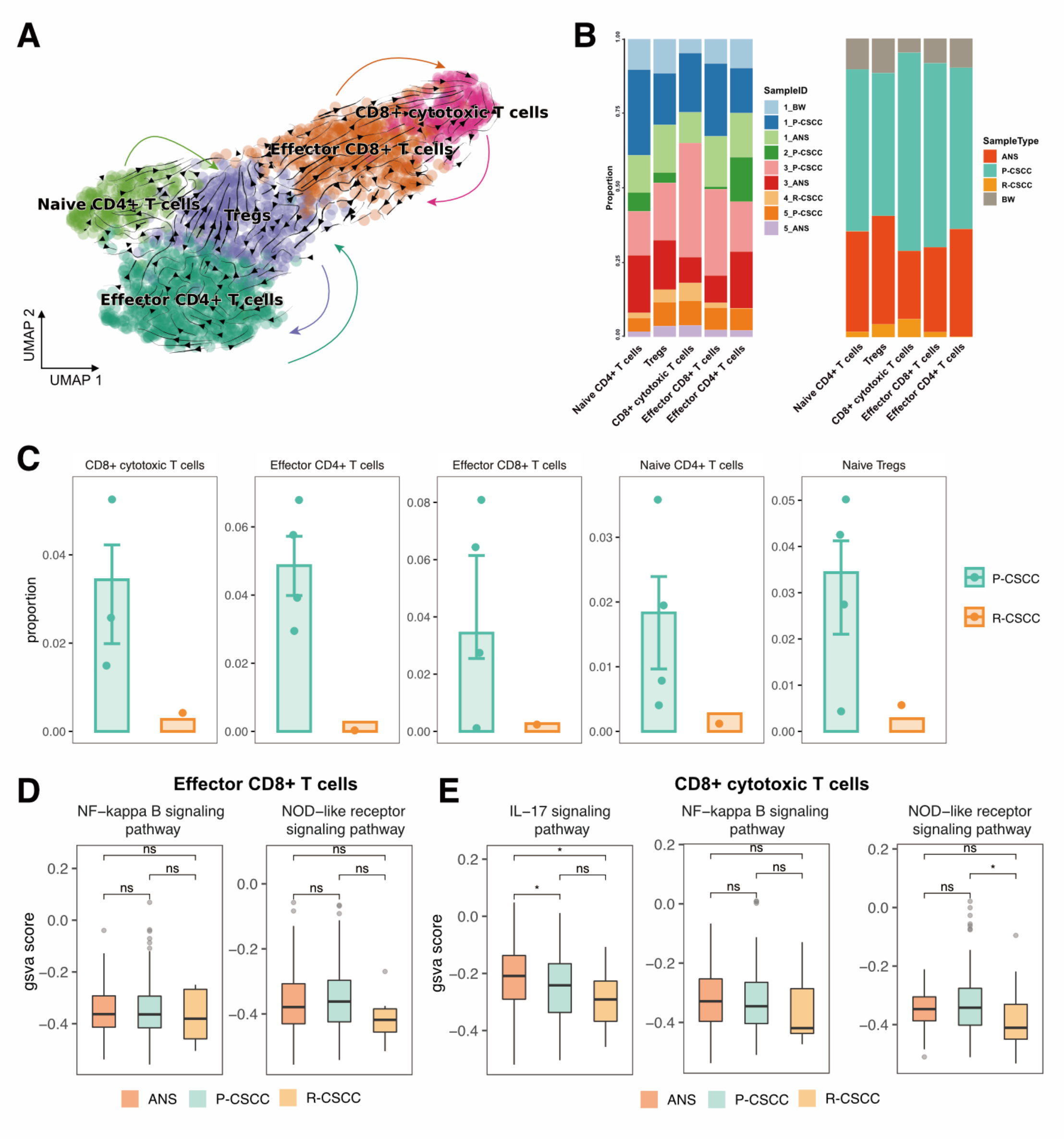

Figure S4. Single-cell transcriptomic analysis of T cells in cSCC. A. Cell transition potential determined by RNA velocity analysis. **B.** The proportion of T cells relative to sample ID and sample type. **C.** Each point represents the proportion of T cells among samples, Y-axis represents the average proportion of T cells in each group, Error bars represent ± S.E.M. **D.** The GSVA score of inflammatory pathways in effector CD8+ T cells among different sample types. **E.** The GSVA score of inflammatory pathways in CD8+ cytotoxic T cells among different sample types. Wilcoxon signed-rank test, $^{*}p < 0.05$.

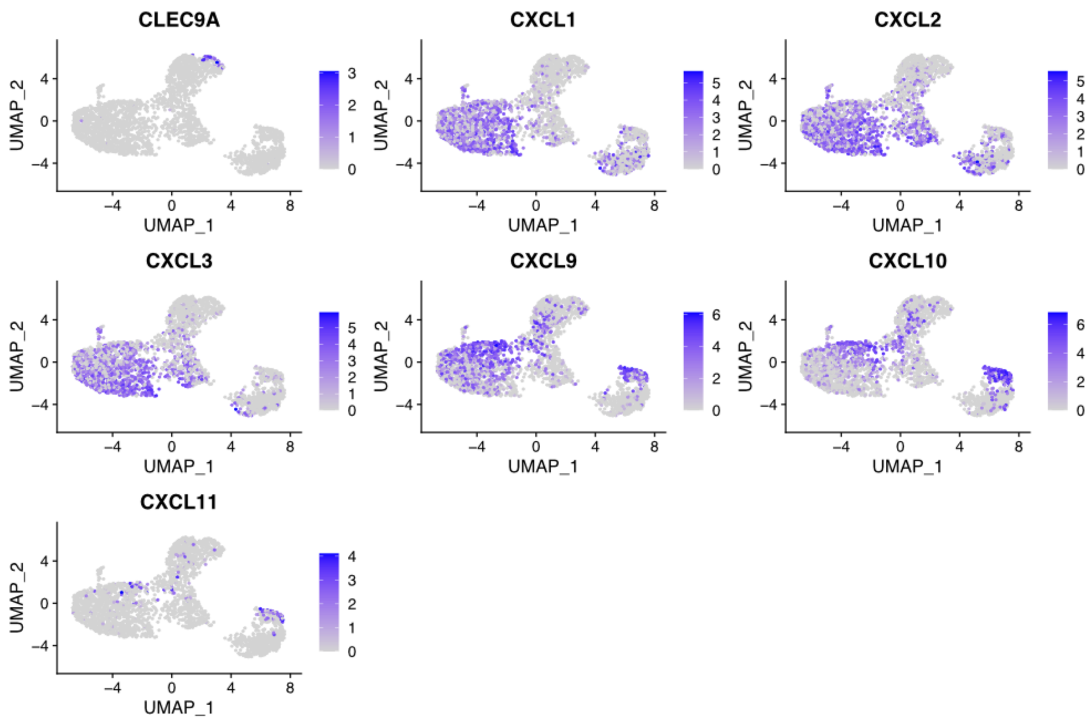

Figure S5. The expression level of gene markers that related with DCs and MDSCs.

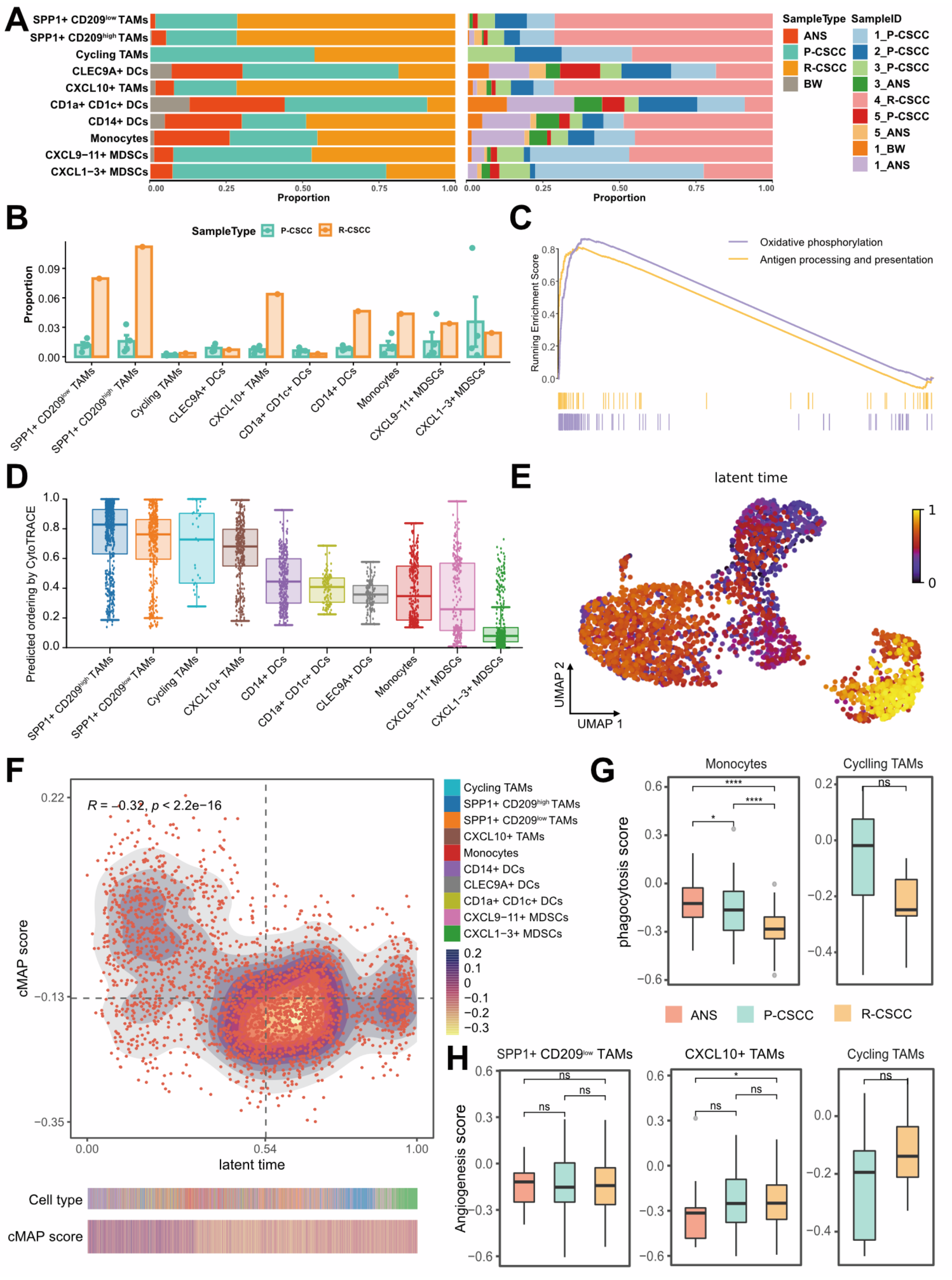

Figure S6. Components and phenotypes of myeloid cells in cSCC. A. The proportion of myeloid cells relative to sample ID and sample type. **B.** Each point represents the proportion of myeloid cells among samples, Y-axis represents the average proportion of myeloid cells in each group, Error bars represent ± S.E.M. **C.** Significant enriched pathways in CXCL9-11^{+} MDSCs versus CXCL1-3^{+} MDSCs. **D.** Distribution of CytoTRACE score in each cell type, ranking by the median value. **E.** UMAP plot

showing the latent time estimated by scVelo tool. **F.** The top scatter plot showing the relationship between latent time and cMAP pathway score, the bottom heatmap plot showing the cMAP score relative to the latent time. **G.** The GSVA score of phagocytosis in monocytes and Cycling TAMs among different sample types. **H.** The GSVA score of angiogenesis in SPP1^{+} CD209^{low} TAMs, CXCL10^{+} TAMs and Cycling TAMs among different sample types. Wilcoxon signed-rank test, $^{*}p < 0.05$, $^{****}p < 0.0001$.

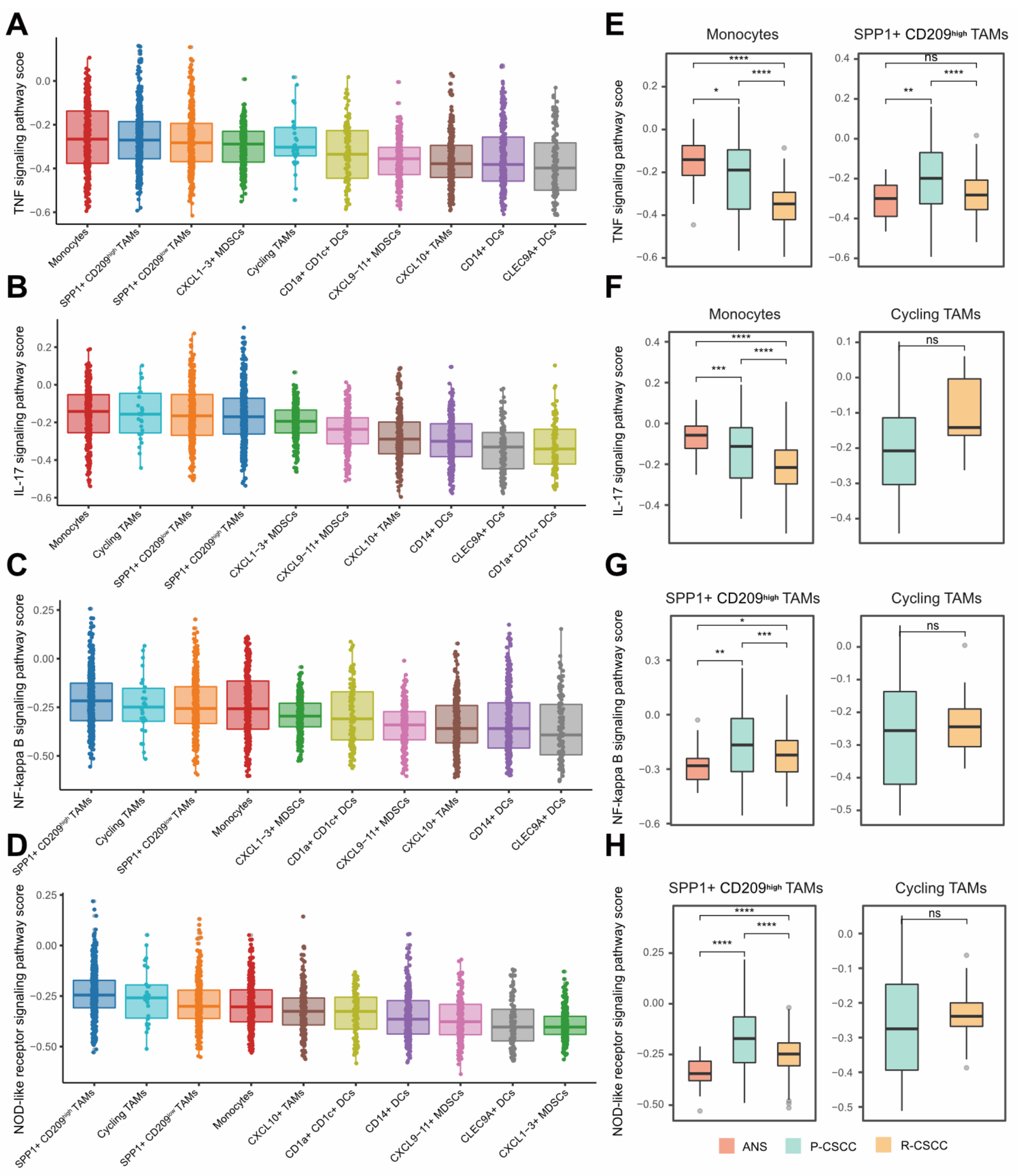

Figure S7. The inflammatory character of myeloid cells in cSCC. A. Distribution of TNF signaling pathway score in each cell type, ranking by the median value. **B.** Distribution of IL-17 signaling pathway score in each cell type, ranking by the median value. **C.** Distribution of NF-kappa B signaling pathway score in each cell type, ranking by the median value. **D.** Distribution of NOD-like receptor signaling pathway score in each cell type, ranking by the median value. **E.** The GSVA score of TNF signaling pathway in monocytes and SPP1^{+} CD209^{high} TAMs among different sample types. **F.** The GSVA score of IL-17 signaling pathway in monocytes and Cycling TAMs among different sample types. **G.** The GSVA score of NF-kappa B signaling pathway in SPP1^{+}

CD209^{high} TAMs and Cycling TAMs among different sample types. **H.** The GSVA score of NOD-like receptor signaling pathway in SPP1^{+} CD209^{high} TAMs and Cycling TAMs among different sample types. Wilcoxon signed-rank test, $^{*}p < 0.05$, $^{**}p < 0.01$, $^{***}p < 0.001$, $^{****}p < 0.0001$.

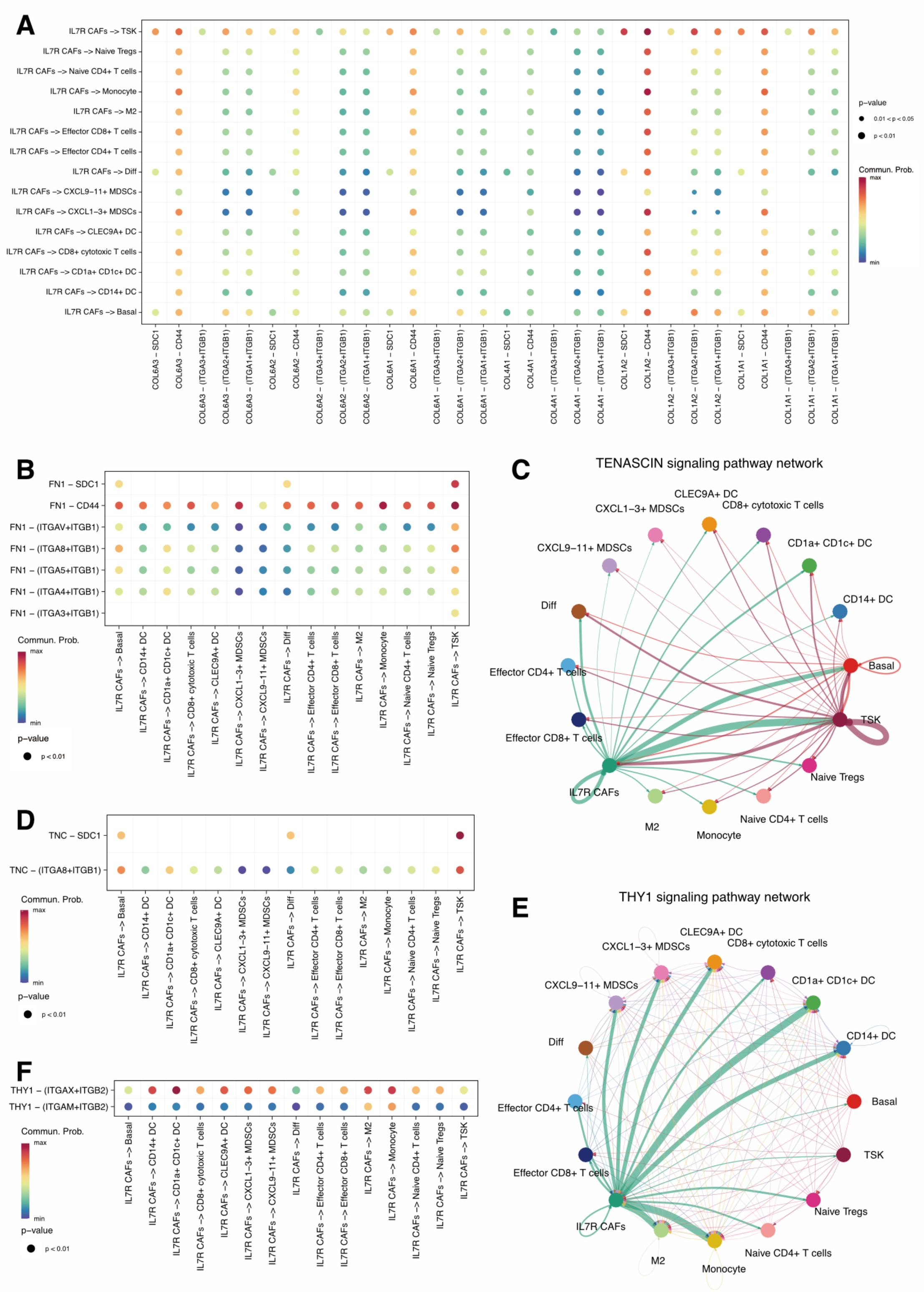

Figure S8. Cell-cell interactions between IL7R⁺ CAFs and other cells. (A, B, D, F) Summary of ligand–receptor interactions of COLLAGEN signaling pathway, FN1 signaling pathway, TENASCIN signaling pathway and THY1 signaling pathway. P–values are represented by the size of each circle. The color gradient indicates the

communication probability of interaction. **(C, E)** Cell-cell interactions in TENASCIN signaling pathway and THY1 signaling pathway.

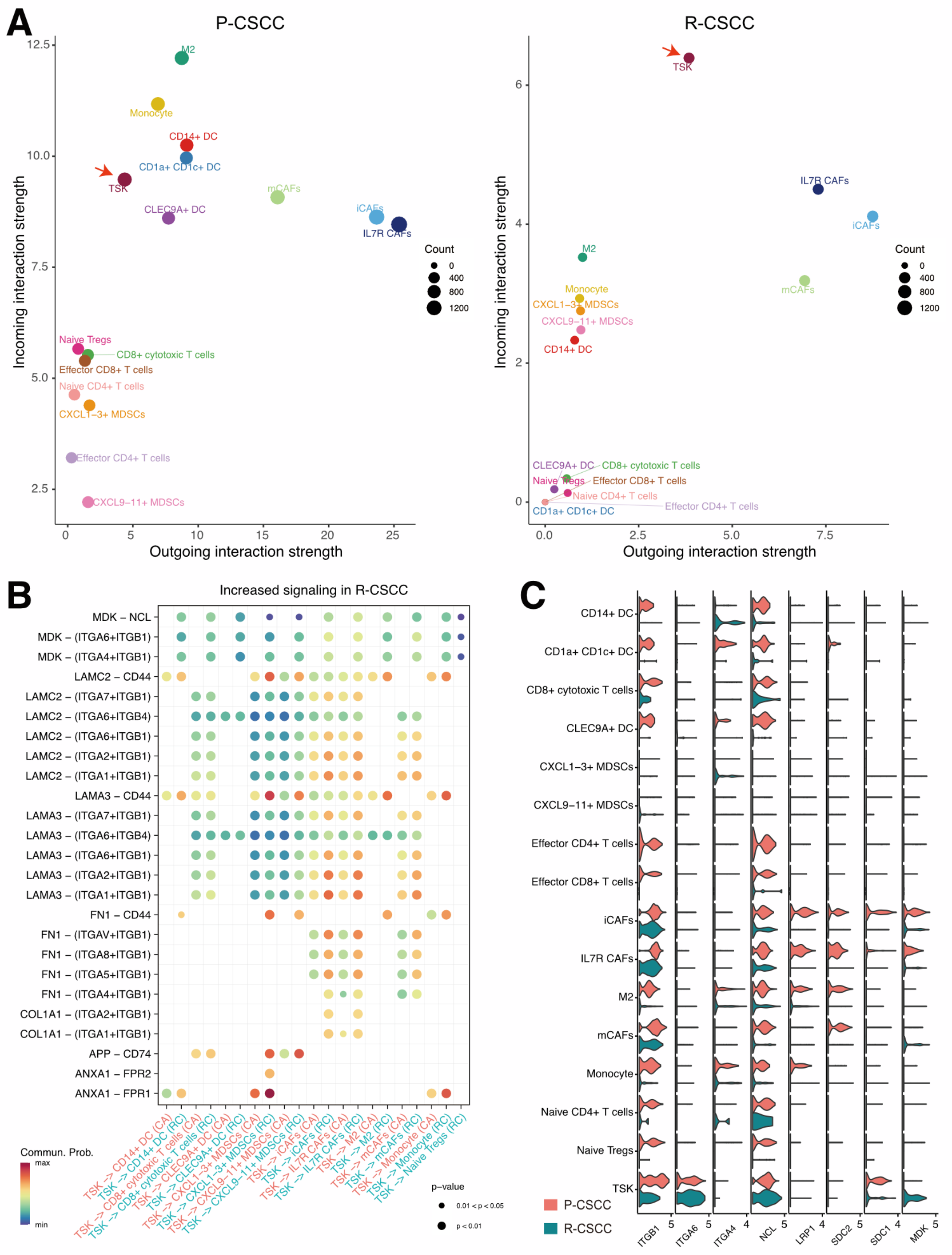

Figure S9. Cell-cell interactions of the primary and recurrent cSCC. A. The incoming and outgoing interaction strength of different cells in primary and recurrent cSCC. **B.** Summary of ligand–receptor interactions of increased signaling pathway in recurrent cSCC. P–values are represented by the size of each circle. The color gradient indicates the communication probability of interaction. **C.** The expression of ligands and receptors among different cells, colors represent different sample types.

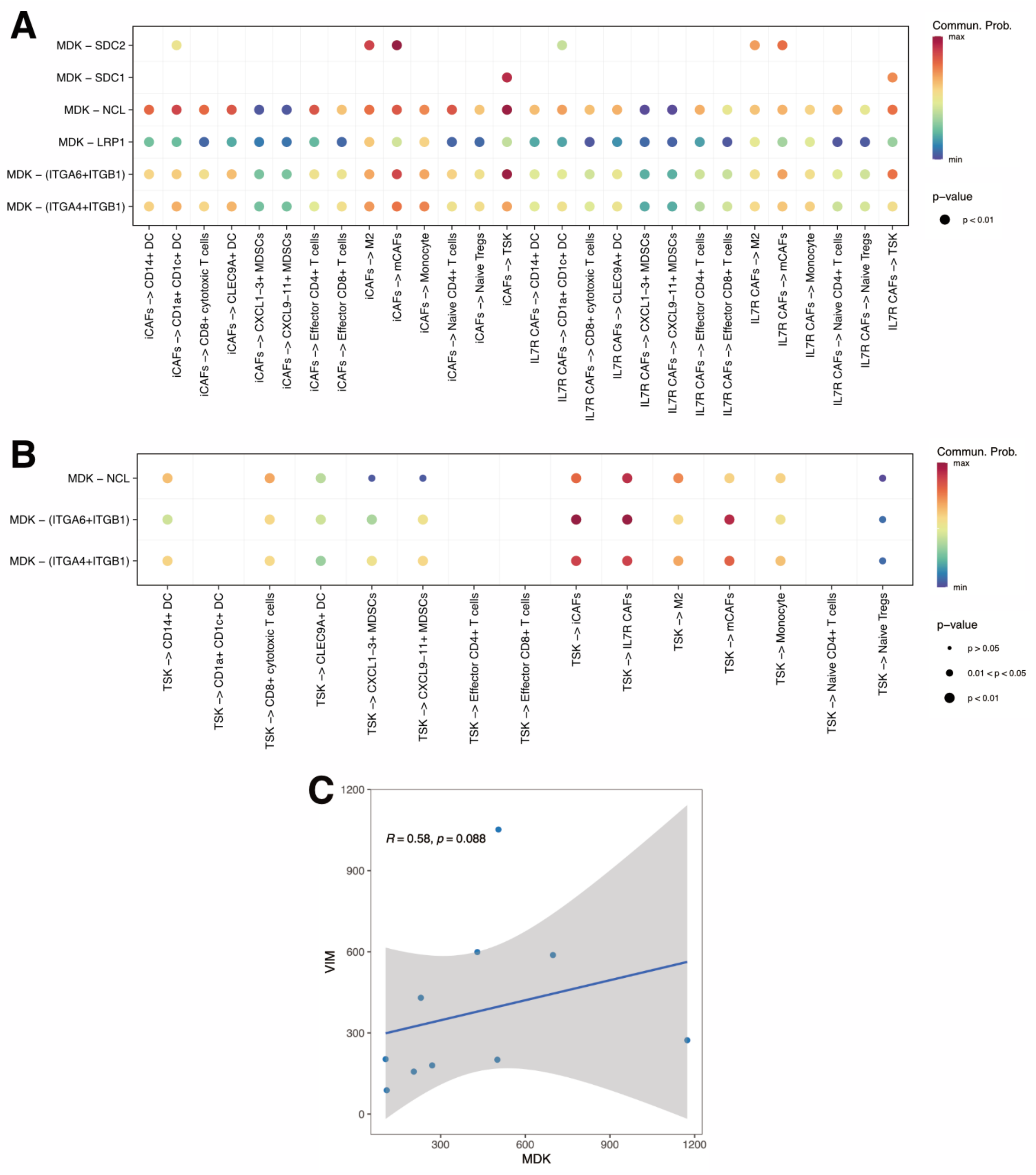

Figure S10. The specific ligand–receptor interactions of MDK signaling pathway within TME and correlation analysis in clinical samples. A. Summary of ligand–receptor interactions of MDK signaling pathway in primary cSCC. **B.** Summary of ligand–receptor interactions of MDK signaling pathway in recurrent cSCC. P–values are represented by the size of each circle. The color gradient indicates the communication probability of interaction. **C.** Scatter plot of the score of MDK and VIM in AK samples.

Table S1. Sample information.

Donor ID	Sample ID	Location	Sex	Age	Sample Type	Treatment
1	1_BW	Skin	Male	65	BW	SSP
1	1_ANS	Skin	Male	65	ANS	SSP
1	1_P-cSCC	Skin	Male	65	P-cSCC	SSP
2	2_P-cSCC	Eyebrow arch	-	-	P_cSCC	SSP
3	3_P-cSCC	Scalp	Male	70	P_cSCC	SSP
3	3_ANS	Scalp	Male	70	ANS	SSP
4	4_R-cSCC	Skin	Male	63	R-cSCC	MMS
5	5_P-cSCC	Foot	Female	58	P-cSCC	SSP
5	5_ANS	Foot	Female	58	ANS	SSP

SSP: standard surgical procedure; MMS: Mohs micrographic surgery

Table S2. IHC score of VIM, TGFB1 and MDK.

Patient ID	Type	VIM (ANS)	VIM (cSCC)	TGFB1 (ANS)	TGFB1 (cSCC)	MDK (ANS)	MDK (cSCC)	Treatment
1	Primary	0	0	0	0	0	0	SSP
1	Recurrence^{1st}	0	2	0	1	1	2	SSP
1	Recurrence^{2nd}	0	3	0	3	0	4	MMS
2	Primary	0	1	0	0	0	0	SSP
2	Recurrence^{1st}	0	3	0	4	0	2	SSP
2	Recurrence^{2nd}	0	3	0	3	1	3	MMS
3	Primary	0	0	0	0	0	1	SSP
3	Recurrence^{1st}	0	2	0	2	0	3	SSP
3	Recurrence^{2nd}	0	2	0	3	0	3	MMS
4	Primary	0	0	0	0	1	2	SSP
4	Recurrence^{1st}	0	1	0	1	0	4	SSP
4	Recurrence^{2nd}	0	2	0	3	0	3	MMS
5	Primary	0	0	0	0	0	1	SSP
5	Recurrence^{1st}	0	3	0	3	0	3	MMS
6	Primary	0	1	0	1	0	0	SSP
6	Recurrence^{1st}	0	3	0	3	0	2	MMS
7	Primary	0	0	0	1	1	2	SSP
7	Recurrence^{1st}	0	2	0	4	0	3	SSP
7	Recurrence^{2nd}	0	3	0	4	1	2	MMS
8	Primary	0	0	0	0	0	1	SSP
8	Recurrence^{1st}	0	2	0	1	0	2	MMS
9	Primary	0	1	0	1	0	0	SSP
9	Recurrence^{1st}	0	3	0	2	0	4	MMS
10	Primary	0	0	0	0	0	1	SSP
10	Recurrence^{1st}	0	4	0	3	0	4	MMS
11	Primary	0	0	0	0	0	0	SSP
11	Recurrence^{1st}	0	3	0	3	0	3	MMS
12	Primary	0	1	0	0	0	0	SSP
12	Recurrence^{1st}	0	3	0	3	0	3	SSP
12	Recurrence^{2nd}	0	2	0	4	0	3	MMS
13	Primary	0	0	0	0	0	1	SSP
13	Recurrence^{1st}	0	2	0	1	0	2	SSP
13	Recurrence^{2nd}	0	4	0	2	1	2	MMS
14	Primary	0	1	0	1	0	2	SSP
14	Recurrence^{1st}	0	3	0	1	0	3	MMS
15	Primary	0	0	0	0	0	0	SSP
15	Recurrence^{1st}	0	2	0	3	0	3	SSP
15	Recurrence^{2nd}	0	3	0	4	0	3	MMS
16	Primary	0	0	0	0	0	0	SSP
16	Recurrence^{1st}	0	2	0	2	1	3	MMS

SSP: standard surgical procedure; MMS: Mohs micrographic surgery

Table S3. IHC score of VIM and MDK in Hypodermis.

Type	VIM (No of positive cell/area)	MDK (No of positive cell/area)
Normal Skin	10	23
Normal Skin	7	42
Normal Skin	6	19
Normal Skin	13	50
Normal Skin	4	17
Actinic Keratosis	180	270
Actinic Keratosis	1052	505
Actinic Keratosis	430	230
Actinic Keratosis	588	698
Actinic Keratosis	273	1174
Actinic Keratosis	599	430
Actinic Keratosis	201	501
Actinic Keratosis	88	109
Actinic Keratosis	157	205
Actinic Keratosis	203	105
